# Diabetic neuropathy: cutting-edge research and future directions

**DOI:** 10.1038/s41392-025-02175-1

**Published:** 2025-04-25

**Authors:** Yang Yang, Bing Zhao, Yuanzhe Wang, Hongli Lan, Xinyu Liu, Yue Hu, Peng Cao

**Affiliations:** 1 State Key Laboratory on Technologies for Chinese Medicine Pharmaceutical Process Control and Intelligent Manufacture, Nanjing University of Chinese Medicine, Nanjing, China; 2 Jiangsu Provincial Medical Innovation Center, Affiliated Hospital of Integrated Traditional Chinese and Western Medicine, Nanjing University of Chinese Medicine, Nanjing, China

**Keywords:** Neurodevelopmental disorders, Diseases of the nervous system

## Abstract

Diabetic neuropathy (DN) is a prevalent and debilitating complication of diabetes mellitus, significantly impacting patient quality of life and contributing to morbidity and mortality. Affecting approximately 50% of patients with diabetes, DN is predominantly characterized by distal symmetric polyneuropathy, leading to sensory loss, pain, and motor dysfunction, often resulting in diabetic foot ulcers and lower-limb amputations. The pathogenesis of DN is multifaceted, involving hyperglycemia, dyslipidemia, oxidative stress, mitochondrial dysfunction, and inflammation, which collectively damage peripheral nerves. Despite extensive research, disease-modifying treatments remain elusive, with current management primarily focusing on symptom control. This review explores the complex mechanisms underlying DN and highlights recent advances in diagnostic and therapeutic strategies. Emerging insights into the molecular and cellular pathways have unveiled potential targets for intervention, including neuroprotective agents, gene and stem cell therapies, and innovative pharmacological approaches. Additionally, novel diagnostic tools, such as corneal confocal microscopy and biomarker-based tests, have improved early detection and intervention. Lifestyle modifications and multidisciplinary care strategies can enhance patient outcomes. While significant progress has been made, further research is required to develop therapies that can effectively halt or reverse disease progression, ultimately improving the lives of individuals with DN. This review provides a comprehensive overview of current understanding and future directions in DN research and management.

## Introduction

Diabetic neuropathy (DN) is among the most prevalent chronic complications of types 1 and 2 diabetes mellitus (T1DM and T2DM), significantly compromising patients’ quality of life and contributing to elevated morbidity and mortality rates.^1,2^ The number of patients with diabetes globally is expected to reach 783 million by 2045.^3^ The diabetes population could reach 1.31 billion by 2050.^4,5^ Diabetic peripheral neuropathy (DPN), the most common form of DN, is characterized by distal symmetric polyneuropathy, leading to sensory loss, pain, and motor dysfunction in the extremities and is a primary cause of foot ulcers and lower-limb amputations in diabetes patients.^1,4,6^ DPN imposes significant economic burdens on healthcare systems.^7,8^ With the rising global prevalence of diabetes driven by aging populations and lifestyle changes, the impact of DN is expected to escalate.^9^ Despite its severity, DN has received comparatively less attention in clinical research and therapeutic development than other diabetic complications, such as nephropathy and retinopathy, necessitating further research.^6,10–12^

The pathogenesis of DN is multifactorial and remains poorly understood.^1,4^ Hyperglycemia, dyslipidemia, and insulin resistance activate various metabolic pathways, resulting in oxidative stress, mitochondrial dysfunction, inflammation, and microvascular damage, culminating in neuronal injury, Schwann cell damage, and myelin sheath degeneration.^13–15^ Additionally, impaired neurotrophic support and autoimmune mechanisms may further exacerbate neuronal degeneration.^16^ Despite extensive research, effective treatments capable of halting or reversing DN’s progression remain elusive. Current management strategies primarily focus on alleviating symptoms and controlling pain.^11^ Antidepressants, anticonvulsants, and opioids have shown limited efficacy and are associated with various side effects, such as dizziness and nausea.^17,18^ Furthermore, no novel therapies have received approval in recent years.^4,11^

Recent research has shifted toward elucidating the molecular pathways involved in DN to identify potential therapeutic targets.^6,11^ Areas of interest include oxidative stress, chronic low-grade inflammation, mitochondrial dysfunction, and central sensitization mechanisms that contribute to neuropathic pain. Early diagnostic markers and advanced imaging techniques, such as corneal confocal microscopy and skin biopsy, enable timely intervention.^1,11,19^ Novel pharmacological agents, including sodium-glucose cotransporter 2 (SGLT2) inhibitors, glucagon-like peptide-1 (GLP-1) receptor agonists, and ion channel blockers, have shown promising neuroprotective and pain management effects.^11,20,21^ Additionally, non-pharmacological approaches, including lifestyle modifications, are being explored to enhance patient outcomes and quality of life.^22,23^

The profound impact of DN on individuals and healthcare systems, combined with the limitations of current treatment options, necessitates further research.^6,15^ This review summarizes recent advances in the pathophysiology of DN, discusses current diagnostic and therapeutic strategies, and highlights potential future directions in managing this debilitating condition.^24^

## Epidemiology of DN

### Global prevalence and trends

Current estimates suggest that approximately 50% of diabetes patients will develop some form of neuropathy.^4^ However, the reported prevalence of DN varies significantly across studies, ranging from 6% to over 60%.^4,25^ The prevalence of DPN is significantly higher in developing countries, often exceeding 50% among diabetes patients.^26^ In a comprehensive study conducted in China, the prevalence of DN among diabetes patients was 37%.^27^ The prevalence of DN is also influenced by age, sex, and ethnicity, with males exhibiting higher rates than females.^5^ Prediabetes is a significant risk factor for DN, with the prevalence of DPN among prediabetes individuals ranging from 6 to 25%.^11^ Compared with those with T1DM, patients with T2DM may present with DN at diagnosis or during the prediabetic state.^28^ In the MONICA/KORA Augsburg surveys, the prevalence of peripheral neuropathy increased from 7.4% in individuals with normal glucose tolerance to 28.0% in those with T2DM as glucose tolerance worsened.^28^ Additionally, the method used to diagnose DN affects the reported prevalence. For example, when using questionnaires like MNSIQ, the prevalence of DN in T1DM patients is estimated at 11–13%;^29,30^ however, when structured clinical examinations and vibration perception thresholds are used, the prevalence increases to 28%.^31,32^ With more sensitive tests, the prevalence exceeds 50%.^33,34^ The number of young individuals diagnosed with T2DM has been increasing.^35^ Geographic and cultural factors also influence the prevalence of DN.^36^ Recent studies have adopted widely recognized definitions of neuropathic pain.^37^ Most reports estimate the prevalence of painful DPN (PDPN) among diabetes patients to be between 13 and 35%.^38,39^ In the MONICA/KORA study, the overall prevalence of neuropathic pain was 13.3% among diabetes patients, 8.7% in those with impaired glucose tolerance, 4.2% in those with impaired fasting glucose, and 1.2% in individuals with normal glucose tolerance.^40^

The increase in diabetes prevalence likely increases the incidence of DN. DN imposes substantial costs due to increased healthcare utilization, lost productivity, and decreased quality of life.^41^ Despite its clinical significance, epidemiological research concerning DN is less extensive than other diabetic complications.^42^ Addressing the gaps in DN epidemiology requires coordinated efforts to improve research methodologies and promote international collaboration to develop effective prevention and treatment strategies.^1^

### Risk factors

The development and progression of DN are influenced by various factors, including the duration of diabetes, glycemic control, age, obesity, dyslipidemia, insulin resistance, chronic low-grade inflammation, lifestyle choices, cardiovascular health, and genetic predispositions.^5,11,43^ Prolonged diabetes leads to cumulative nerve damage.^44,45^ Elevated glycated hemoglobin (HbA1c) levels are associated with increased neuropathy risk.^13,46^ Intensive glycemic control significantly reduces the incidence and progression of neuropathy in T1DM and T2DM patients.^47,48^ Age-related degenerative changes make older adults more susceptible to nerve damage, with DN prevalence significantly increasing in individuals over 50 years old.^1,49^

Obesity and dyslipidemia are major metabolic risk factors; a higher body mass index (BMI) and dyslipidemia promote insulin resistance and metabolic dysfunction, contributing to neuropathy development.^1,50–52^ Insulin resistance, even without overt hyperglycemia, independently increases DN risk by causing peripheral nerve damage.^53,54^ Chronic low-grade systemic inflammation elevates levels of C-reactive protein (CRP) and proinflammatory cytokines, leading to endothelial dysfunction and nerve injury.^55^ Lifestyle factors such as smoking, excessive alcohol consumption, and physical inactivity are linked to DN development, exacerbating oxidative stress and vascular dysfunction.^22,56,57^ Cardiovascular diseases and related risk factors (e.g., hypertension, atherosclerosis) impair blood flow to peripheral nerves, worsening neuropathic symptoms.^33,58^ Genome-wide association studies (GWAS) have identified genetic variants related to metabolic regulation, nerve repair, and vascular function.^59,60^ Specific genes implicated include vascular endothelial growth factor (VEGF), neurotrophic receptor tyrosine kinase 1, sodium voltage-gated channel alpha subunit 2 (SCN2A), angiotensin-converting enzyme, and methylenetetrahydrofolate reductase, with polymorphisms associated with increased DN susceptibility.^61–64^ Additionally, emerging evidence suggests that virus infections, such as COVID-19, exacerbate neuropathies in diabetes patients through inflammatory and immune-mediated mechanisms.^65–67^

**Fig. 1:**
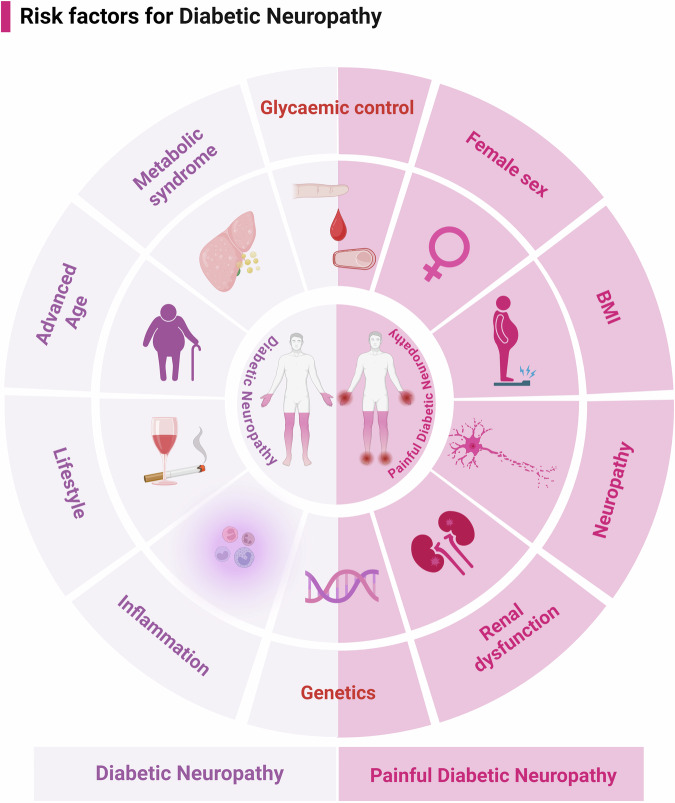
Risk factors for diabetic neuropathy. The development and progression of DN are influenced by multiple factors, including diabetes duration, poor glycemic control, advanced age, and MetS, which encompasses obesity, hypertension, and dyslipidemia. Other contributing factors include chronic low-grade inflammation, lifestyle choices such as smoking and alcohol abuse, and genetic predisposition. Risk factors for painful diabetic neuropathy are less well-defined, but overlap with those for diabetic nephropathy. These include poor blood glucose control, susceptibility genes, and independent factors such as female sex, increased neuropathy severity, renal dysfunction, and higher BMI. BMI body mass index, DN diabetic neuropathy, MetS metabolic syndrome

Although the risk factors for PDPN are poorly understood, female sex, longer diabetes duration, increased neuropathy severity, renal dysfunction, higher HbA1c levels, and higher BMI have been suggested as contributing factors.^68–74^ Genetic factors have also been suggested; gain-of-function mutations in voltage-gated sodium channel genes increase neuronal excitability and are associated with PDPN.^75,76^ GWAS have identified single nucleotide polymorphisms near genes GFRA2, HMGB1P46, and ZSCAN20-TLR12P in patients with PDPN (Fig. 1).^77,78^ Comprehensive research on the risk factors for DN is limited, particularly concerning the genetic and viral infection influences on PDPN. Future studies should explore these complex factors to develop more effective prevention and treatment strategies for better disease management goals.

## Classification of DN

**Fig. 2:**
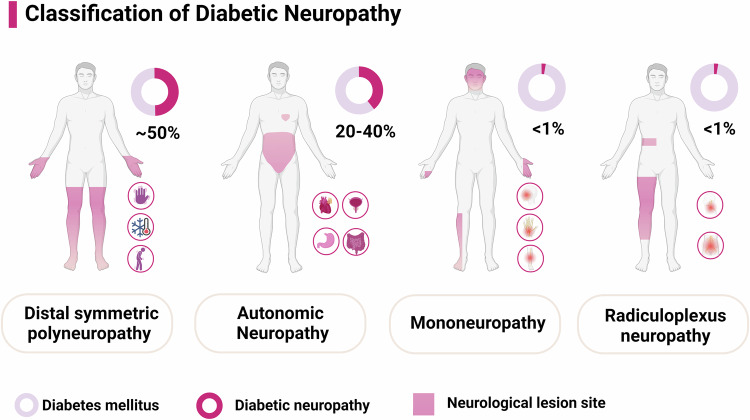
Classification of diabetic neuropathy. Diabetic neuropathies comprise a diverse group of clinical syndromes, typically classified by the pattern of neurological involvement. Distal symmetric polyneuropathy (DSPN) is the most common form, which affects approximately 50% of patients with diabetes, characterized by sensory loss, pain, and motor dysfunction in the extremities. Autonomic neuropathy, affecting 20–40% of patients with diabetes, primarily involves the autonomic nervous system, impacting organs such as the heart, gastrointestinal tract, and urogenital system. Mononeuropathy, with an incidence of < 1%, usually involves isolated cranial nerves (e.g., oculomotor, facial) or peripheral nerves (e.g., facial). Radiculoplexus neuropathy, also occurring in <1% of diabetes cases, predominantly affects the lumbar or cervical plexus. DSPN distal symmetric polyneuropathy, DM Diabetes mellitus

DN encompasses a heterogeneous group of disorders affecting various components of the peripheral nervous system (PNS) in patients with diabetes.^1,4,11,41,79^ Classification is essential for understanding the diverse manifestations and guiding appropriate management strategies (Fig. 2).

### DPN

Diffuse neuropathies are the most common forms of DN, primarily represented by distal symmetric polyneuropathy (DSPN), also known as DPN.^4^ DPN affects approximately 50% of individuals with diabetes during their lifetime and can be classified based on the type of nerve fibers affected.^4,44^

#### Small fiber neuropathy (SFN)

SFN primarily affects small unmyelinated C fibers and thinly myelinated Aδ fibers, leading to disturbances in pain and temperature sensation.^80,81^ The overall prevalence of SFN in diabetes patients ranges from 20 to 30%, depending on the diagnostic criteria and population studied.^1,4^ Painful DN, often associated with SFN, occurs in approximately 25% of diabetes patients and significantly impacts the quality of life due to chronic pain and sensory disturbances.^1,4^ Early symptoms often include painful sensations, such as burning pain, electric shock-like sensations, sharp pains, and hyperalgesia.^82,83^ Patients may also experience paresthesia and allodynia, where non-painful stimuli are perceived as painful.^84,85^ Recent studies highlight the importance of SFN in DN and its potential role as an early indicator of neuropathic complications.^1,86^

#### Large fiber neuropathy

Large fiber neuropathy affects larger myelinated Aβ fibers, resulting in sensory and motor deficits.^1^ The prevalence of large fiber neuropathy in diabetes patients is 40–60%.^87,88^ Sensory deficits include reduced proprioception and vibration sense, leading to numbness and balance difficulties.^1,89^ Motor deficits manifest as muscle weakness and atrophy in severe cases, as well as loss of deep tendon reflexes, particularly the ankle reflex.^90,91^ These impairments increase the risk of falls and foot ulcers due to unnoticed injuries.^92,93^ Advances in neurophysiological assessments, such as nerve conduction studies (NCS), have improved the detection of large fiber involvement in DN.^89,94^

#### Mixed fiber neuropathy

In many patients, small and large fibers are affected, leading to a combination of sensory deficits and neuropathic pain.^1,72^ The prevalence of mixed fiber neuropathy is substantial, with up to 50%.^26,95^ Clinical manifestations include symmetrical sensory loss typically initiated in the toes and progresses proximally in a “stocking-glove” distribution.^1^ Neuropathic pain is present in a significant subset of patients.^83^ Involvement of autonomic fibers can lead to sweating abnormalities, orthostatic hypotension, and gastrointestinal disturbances.^1^ Early detection and comprehensive management are crucial to prevent complications.^26,95^

#### Diagnosis and screening recommendations

Early detection of DPN is crucial to prevent foot ulcers and amputations, among other complications.^5,96^ Effective diagnosis and screening involve a combination of clinical assessments, sensory testing methods, electrophysiological studies, and advanced diagnostic methods tailored to the specific neuropathy subtype.^80,97–101^ A detailed history taking identifies symptoms related to sensory, motor, and autonomic dysfunction, while a physical examination focuses on sensory and motor deficits, and reflex abnormalities.^44,102^ Sensory testing methods include the 10 g monofilament test for pressure perception,^103^ the 128 Hz tuning fork for vibration sense,^35^ pinprick and temperature sensation tests for small fiber function,^104^ and loss or reduction of ankle reflexes indicating large fiber involvement.^91^ NCS is recommended for atypical presentations or to exclude other causes of neuropathy.^79,105^ Advanced diagnostic methods involve skin biopsy for intraepidermal nerve fiber density (IENFD),^87,104^ quantitative sensory testing (QST) for temperature and pain thresholds, corneal confocal microscopy for early small fiber damage,^19,106^ and MRN for detailed imaging of nerve structures.^107^

#### Screening guidelines and components

For T2DM, screening for DPN should begin at diagnosis and occur annually;^79,108^ conversely, for T1DM, screening should start five years after diagnosis and continue annually;^79,108^ individuals with prediabetes exhibiting neuropathic symptoms should be screened;^109^ high-risk patients should be prioritized for screening.^110,111^ Screening components include routine foot examinations for ulcers and abnormalities, patient education on foot care and early signs of neuropathy, and management of modifiable risk factors such as hypertension, dyslipidemia, and smoking. Regular screening and education are essential components of comprehensive care.^110,111^

### Diabetic autonomic neuropathy (DAN)

DAN is a serious and prevalent complication of diabetes originating from damage to the autonomic nervous system (ANS), which governs involuntary bodily functions. The overall prevalence of DAN varies, ranging from 20 to 40% among diabetes patients.^112^ DAN encompasses multiple subtypes, including cardiovascular autonomic neuropathy (CAN), gastrointestinal autonomic neuropathy, genitourinary autonomic neuropathy, sudomotor dysfunction, hypoglycemia unawareness, and pupillary dysfunction, each affecting different organ systems and presenting a spectrum of clinical manifestations.

#### CAN

CAN is one of the most clinically significant forms of DAN due to its strong association with increased morbidity and mortality. The prevalence of CAN among diabetes patients ranges from 20 to 60%.^113,114^ Early stages of CAN may be asymptomatic and detectable only through reduced heart rate variability (HRV) during deep breathing.^115^ As CAN progresses, patients may experience resting tachycardia, exercise intolerance, orthostatic hypotension, and silent myocardial ischemia.^116^

Diagnostic approaches for CAN include HRV tests such as the deep breathing test, valsalva maneuver, and orthostatic testing, along with ambulatory blood pressure monitoring to identify irregular circadian blood pressure patterns.^117–120^ Recent advancements have introduced novel biomarkers and advanced imaging techniques to enhance early detection.^114,121^

#### Gastrointestinal autonomic neuropathy

Gastrointestinal autonomic neuropathy presents with various symptoms, such as esophageal dysmotility, gastroparesis, and enteropathy, including dysphagia, gastroesophageal reflux, delayed gastric emptying, diarrhea, constipation, and fecal incontinence.^122–124^ The prevalence of gastrointestinal autonomic neuropathy in diabetes patients is estimated to be between 30 and 50%.^124,125^

Key diagnostic approaches include gastric emptying scintigraphy, the 13C-octanoic acid breath test, wireless motility capsule for gastroparesis, and esophageal manometry with pH monitoring for esophageal dysmotility.^126–130^ Recent innovations, such as Gastric Per-Oral Endoscopic Myotomy (G-POEM) for refractory gastroparesis and advancements in enteric nervous system research, aim for targeted therapies.^125,131,132^

#### Genitourinary autonomic neuropathy

Genitourinary autonomic neuropathy involves bladder dysfunction, such as decreased bladder sensation, urinary retention, recurrent urinary tract infections, and incontinence, alongside sexual dysfunction.^133–137^ The prevalence of genitourinary autonomic neuropathy is up to 40% in diabetes patients, with erectile dysfunction (ED) affecting up to 75% of diabetic males due to vascular and neuropathic factors.^135,136^ In females, symptoms include decreased libido, vaginal dryness, dyspareunia, and reduced sexual arousal, with prevalence of 30–50%.^138^

Diagnostic approaches encompass post-void residual volume measurement, urodynamic studies, comprehensive medical and sexual history assessments, nocturnal penile tumescence, penile Doppler ultrasonography for males, and detailed sexual function histories and hormonal evaluations for females.^139–141^ Recent advancements have aided in restoring autonomic nerve function.^142,143^

#### Sudomotor dysfunction

Sudomotor dysfunction is characterized by anhidrosis or hyperhidrosis, leading to dry skin or compensatory excessive sweating, respectively, which can increase infection risks.^144^ The prevalence of sudomotor dysfunction in diabetes patients is approximately 30%.^145^

Diagnostic approaches include the Quantitative Sudomotor Axon Reflex Test, Electrochemical Skin Conductance (ESC) using devices like Sudoscan, and the Neuropad Test.^146–148^ Recent developments feature Sudoscan as a reliable early detection method and advanced skin biopsy techniques to assess IENFD.^145,149,150^

#### Hypoglycemia unawareness

Hypoglycemia unawareness impairs counterregulatory hormonal responses during hypoglycemia, which reduces symptom awareness and heightens the risk of severe hypoglycemic episodes.^151,152^ The prevalence of hypoglycemia unawareness among individuals with diabetes on intensive insulin therapy can be as high as 30–40%.^114,152^ Clinically, patients may not recognize hypoglycemia until neuroglycopenic symptoms manifest, such as confusion or seizures.^153^

Diagnostic approaches focus on identifying impaired counterregulatory responses and may include continuous glucose monitoring systems to detect unrecognized hypoglycemic events.^154^ Recent advancements involve Hybrid Closed-Loop Systems for automated insulin delivery and structured education programs for restoring hypoglycemia awareness.^114,155,156^

#### Pupillary dysfunction

Pupillary dysfunction manifests as miosis, reduced light reflex, and difficulties in adapting to lighting changes.^157,158^ The prevalence of pupillary dysfunction in diabetes patients is around 25%.^125,157^ Diagnostic approaches include pupillometry for objectively measuring pupil size and reactivity, pharmacological testing to assess responses to agents like pilocarpine, and dynamic pupillometry as a non-invasive biomarker for autonomic dysfunction.^125,157,159^ Recent progress encompasses the application of Artificial Intelligence (AI) algorithms to analyze pupillary responses for early detection.^125,157,159^

Recent studies on DAN have advanced diagnostic techniques and subtype classification; however, the comprehension of its pathophysiological mechanisms and effective treatment strategies remains inadequate. It is necessary to strengthen studies on various types of autonomic neuropathy, develop more precise diagnostic tools, and explore personalized treatment plans to improve patients’ quality of life.

### Mononeuropathy

Mononeuropathy refers to the damage of a single or a group of nerves, resulting in localized symptoms such as pain, weakness, or sensory loss in the affected area. Diabetes patients are more susceptible to mononeuropathies compared to non-diabetes individuals.^24,41^ Although mononeuropathies are less common than other forms of DN, with a prevalence estimated to be below 1% among diabetes patients,^44,160^ and are more frequently observed in older individuals and those with a longer duration of diabetes.^42^ Commonly affected nerves include the cranial nerves.^161^ The facial nerve (cranial nerve VII) may present with facial paralysis or weakness, and less commonly, the trochlear (CN IV), trigeminal (CN V), and abducens (CN VI) nerves can be involved.^41,162^ Peripheral nerves commonly affected include the median nerve, the ulnar nerve, the radial nerve, and the common peroneal (fibular) nerve.^163–165^ Multiple mononeuropathy, or mononeuritis multiplex, involves two or more non-contiguous nerve trunks and presents with asymmetric, stepwise progression of symptoms, necessitating differentiation from more symmetrical polyneuropathy.^166,167^ The pathophysiology of diabetic mononeuropathy includes ischemic mechanisms, such as the occlusion of the vasa nervorum leading to nerve infarction, metabolic factors, including hyperglycemia-induced oxidative stress, and the formation of advanced glycation end products, contributing to nerve damage.^13,168^

Diagnosis involves a comprehensive clinical evaluation, including detailed neurological examination, electrophysiological studies like NCS and electromyography (EMG) to assess motor and sensory fiber function and localize lesions, as well as imaging studies such as magnetic resonance imaging (MRI) to exclude compressive or structural causes.^41,169,170^ Differential diagnoses include entrapment neuropathies, infectious neuropathies, and inflammatory conditions.^167,171,172^ Management strategies focus on tight glycemic control to prevent further nerve damage, symptomatic treatment with analgesics for pain management, physical therapy to maintain muscle strength and prevent contractures, and surgical interventions for entrapment neuropathies when necessary.^17,173,174^ The prognosis for diabetic mononeuropathy is generally favorable, which can significantly improve patient outcomes.^175^ Recent advancements include the development of neuroprotective agents^114,176^ with potentially profound implications for patient care. These agents may help to delay or prevent nerve damage progression, potentially improving quality of life by reducing pain and preserving motor functions in affected patients. Integrating these neuroprotective treatments into standard care protocols could reduce the frequency of interventions, such as surgeries or advanced physical therapy, thus improving the efficacy and efficiency of patient care in clinical settings.

### Radiculoplexus neuropathy

Radiculoplexus neuropathy is a rare disabling complication of diabetes mellitus characterized by damage to nerve roots and plexuses, most commonly affecting the lumbosacral plexus and, less frequently, the cervical plexus.^24,41^ Although DRPN has an estimated prevalence of < 1% among diabetes populations,^177–179^ it predominantly occurs in patients with T2DM, especially in middle-aged and older adults.^178^ Clinically, DRPN presents with a sudden or subacute onset of unilateral, severe pain in the hip, thigh, or buttock, often preceding motor weakness by weeks.^180,181^ Motor symptoms include progressive weakness and atrophy of proximal lower-limb muscles, leading to difficulty rising from a seated position or climbing stairs, accompanied by muscle fasciculations and hyporeflexia.^182,183^ Systemic features, such as significant weight loss, are reported in up to 60% of patients.^184,185^

The pathophysiology of DRPN involves ischemic injury due to microvasculitis leading to nerve fiber ischemia and immune-mediated mechanisms, as evidenced by inflammatory infiltrates in nerve biopsies.^15,186^ Diagnosis is primarily based on clinical evaluation supported by electrophysiological studies.^187,188^ Laboratory tests may reveal elevated inflammatory markers, including erythrocyte sedimentation rate or CRP, and nerve biopsy, though rarely performed, can demonstrate microvasculitis and perivascular inflammatory infiltrates.^189^

Management of DRPN focuses on optimizing glycemic control to prevent further nerve damage.^17,173,184,190^ The prognosis for DRPN is generally favorable, with many patients experiencing significant recovery over months to years, although some may have residual weakness, particularly with early diagnosis and appropriate management.^191^ There have been several advancements in DRPN research, including exploring neuroprotective agents.^192,193^ Current research on DRPN is limited due to its rarity; therefore, data on its pathophysiological mechanisms are scarce. Diagnostic and treatment methods warrant further optimization. Research on this condition remains necessary to develop more effective diagnostic tools and treatment strategies to improve patient outcomes.

Diabetic myelopathy is a rare condition characterized by spinal cord dysfunction in diabetes patients without other identifiable causes, presenting with progressive spastic paraparesis, sensory disturbances below the lesion level, and potential bladder and bowel dysfunction.^194^ The pathophysiology is thought to involve ischemia due to microangiopathy and metabolic disturbances.^195^ Diagnosis typically relies on an MRI of the spine, which may reveal spinal cord atrophy or signal changes and the exclusion of compressive lesions.^114^ Evoked potentials may also be utilized to detect delayed conduction times.^196^ Management primarily focuses on optimal glycemic control and symptomatic treatment.^5^

Additionally, central nervous system (CNS) complications are more prevalent in individuals with diabetes, with up to 50% experiencing some degree of cognitive decline.^197,198^ Cognitive impairment in diabetes is attributed to chronic hyperglycemia leading to cerebrovascular disease, neurodegeneration, and inflammation.^196,199^ Management strategies include intensive glycemic control to potentially slow cognitive decline, as well as lifestyle interventions to support cognitive health.^200,201^ Furthermore, mood disorders are significantly more common in diabetes patients, adversely affecting glycemic control and increasing the risk of complications.^202^ Management of these mood disorders involves psychological interventions such as cognitive behavioral therapy (CBT) and pharmacotherapy with antidepressants, considering their metabolic effects.^173,203^ Understanding and addressing spinal and CNS involvement in DN is crucial for comprehensive patient care.^5^

## Pathophysiology of DN

DN is a multifaceted neurodegenerative disorder resulting from the interplay of metabolic, vascular, and neurodegenerative processes primarily driven by chronic hyperglycemia. Chronic high blood sugar levels initiate a cascade of metabolic abnormalities, which ultimately induce oxidative stress and inflammation, exacerbating nerve injury through excessive production of reactive oxygen species (ROS) and promoting neuronal apoptosis.^4,11,15^ Inflammatory responses mediated by proinflammatory cytokines and pathways, such as nuclear factor kappa B (NF-κB) and Toll-like receptor 4 (TLR4), worsen the condition by amplifying oxidative stress and hindering neuronal repair.^13,53,204–206^ Disturbances in lipid metabolism contribute to neuronal apoptosis, while insulin resistance and deficiencies in neurotrophic factors impede nerve regeneration.^4,207,208^ The resultant neuronal hyperexcitability and microglial activation lead to chronic neuropathic pain.^206,209–212^

DN preferentially targets sensory and autonomic axons, with motor axons affected to a lesser extent. The condition is characterized by the “dying back” of distal sensory axons in a length-dependent manner, presenting as a “stocking and glove” pattern of sensory loss.^4,44^ Schwann cells are also targeted by chronic hyperglycemia, leading to demyelination features in severe cases.^210,213^ Damage to Schwann cells affects axonal support, including cytoskeletal properties and axon-Schwann cell interactions essential for intra-axonal mRNA translation.^214–217^ Additionally, sensory neurons in the dorsal root ganglia (DRG) exhibit altered phenotypes, such as reduced neurofilament synthesis, endoplasmic reticulum stress, and changes in key plasticity molecules, such as growth-associated protein 43 (GAP43) and heat shock proteins, leading to mitochondrial dysfunction and loss of peripheral nerve function.^13,15,218,219^ Recent studies have demonstrated alterations in mRNA and microRNA expression in DRG neurons exposed to chronic diabetes, highlighting the upregulation of pathways involved in inflammation, bioenergetics, and lipid processing.^33,220–222^ These insights facilitated studies on targeted therapies.^33,206,211,222,223^

### DPN pathogenesis

DPN is a complex neurodegenerative disorder resulting from the interplay of hyperglycemia-induced metabolic pathways, oxidative stress, inflammation, and dyslipidemia. These metabolic disturbances activate several pathogenic pathways that contribute to neuronal dysfunction and death, ultimately manifesting as DPN.^1,15^

#### Hyperglycemia-induced metabolic pathways

##### Polyol pathway activation

In a hyperglycemic state, excess glucose is shunted into the polyol pathway, where aldose reductase catalyzes its reduction to sorbitol, which in turn is oxidized to fructose by sorbitol dehydrogenase. This diversion leads to the accumulation of sorbitol and fructose within cells, increasing intracellular osmolarity and causing osmotic stress.^224,225^ The consumption of NADPH in this pathway reduces its availability for regenerating glutathione, thereby increasing oxidative stress.^13,225^ The resultant oxidative damage impairs nerve conduction and leads to neuronal apoptosis. This excess pathway activity causes oxidative stress and Na^+^/K^+^-ATPase dysfunction. These effects result in edema, demyelination, and peripheral nerve necrosis, impairing nerve conduction.^15,226,227^

Recent therapeutic approaches focus on aldose reductase inhibitors (ARIs), such as epalrestat and ranirestat, which have shown promise in reducing sorbitol accumulation and ameliorating neuropathic symptoms in clinical trials.^228,229^ Genetic studies suggest that polymorphisms in the aldose reductase gene influence individual susceptibility to DPN,^230^ highlighting the potential for personalized therapies.

##### Hexosamine pathway

Excess glucose not metabolized via glycolysis can enter the hexosamine pathway, which leads to increased production of uridine diphosphate N-acetylglucosamine (UDP-GlcNAc), which is used for O-linked N-acetylglucosamine (O-GlcNAc) modification of proteins.^231,232^ These modifications alter gene expression and protein function, causing vascular dysfunction, inflammation, and oxidative stress, all of which contribute to the pathogenesis of DPN.^224,233^ Benfotiamine, a lipid-soluble thiamine derivative, has been shown to activate transketolase activity, thereby redirecting glycolytic intermediates away from the hexosamine pathway.^234^ Benfotiamine may improve neuropathic symptoms by reducing UDP-GlcNAc production; however, its long-term efficacy remains uncertain.^224,235^ Currently, no specific inhibitors of the hexosamine pathway have been clinically validated to slow DPN progression, underscoring the need for novel therapeutic agents.^236^

##### Advanced glycation end products (AGEs) formation

Chronic hyperglycemia promotes the non-enzymatic glycation of proteins, lipids, and nucleic acids, leading to the formation of AGEs.^237,238^ AGEs accumulate in peripheral nerves and interact with the receptor for AGEs (RAGE) on cell surfaces, activating intracellular signaling pathways involving NF-κB.^239,240^ This activation increases the expression of proinflammatory cytokines and adhesion molecules, contributing to oxidative stress, inflammation, and vascular dysfunction.^238,241^ Therapeutic strategies targeting AGEs include using AGE inhibitors, such as aminoguanidine, which prevents AGE formation.^239,241^ RAGE antagonists are being explored to block the downstream effects of AGE-RAGE interactions to reduce inflammation and oxidative stress in DPN.^236,238^

##### Protein kinase C pathway

**Fig. 3:**
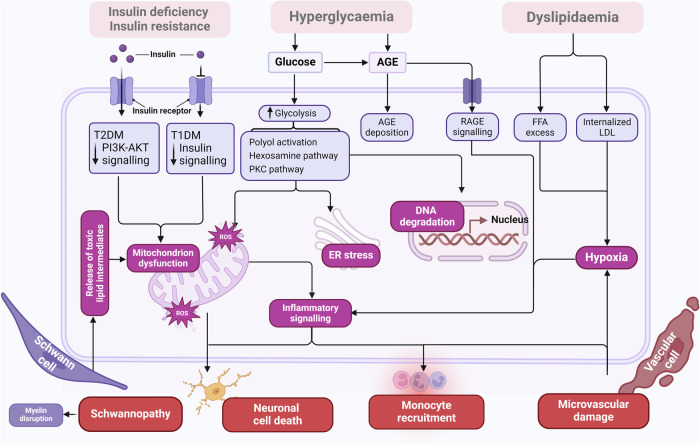
Pathogenesis of diabetic neuropathy. The cellular mechanisms underlying DN involve hyperglycemia, dyslipidemia, and altered insulin signaling. In diabetes mellitus, these factors drive excessive activation of the polyol, hexosamine, and PKC pathways, as well as receptors for RAGE activation. In T1DM, insulin deficiency leads to reduced insulin signaling, while in T2DM, insulin resistance results in impaired PI3K-AKT signaling. These disruptions, along with hyperlipidemia and dyslipidemia, either individually or in combination, contribute to pathological changes in neurons, Schwann cells, and vascular cells, leading to nerve dysfunction and neuropathy. Key pathological processes include DNA damage, ER stress, mitochondrial dysfunction, oxidative stress, neurodegeneration, loss of neurotrophic signaling, and microvascular dysfunction. These changes can also trigger inflammatory and immune-mediated neurotoxicity. The relevance of each pathway to neuropathy development varies based on the cell type, disease profile, and stage, as different cells exhibit varying susceptibility to metabolic dysfunction. AGE advanced glycation end-product, DAG diacylglycerol, ER endoplasmic reticulum, FFAs free fatty acids, GLUT glucose transporters, G-3-P glucosamine 3-phosphate, G-6-P glucosamine 6-phosphate, LDL low-density lipoprotein, LOX-1 oxidized LDL receptor-1, PKC protein kinase C, RAGE AGE-specific receptor, ROS reactive oxygen species, TLR4 Toll-like receptor 4, T1DM type 1 diabetes mellitus, T2DM type 2 diabetes mellitus, UDP-GlcNAc uridine diphosphate N-acetylglucosamine

Protein kinase C (PKC) is involved in intracellular signaling. PKC activation disrupts normal metabolism, leading to ATPase phosphorylation and metabolic disorders, including changes in VEGF and vasoconstriction, which affect pain perception.^242^ PKC activation enhances excitatory currents in the dorsal horn and trigeminal neurons, contributing to nociceptive hypersensitivity in DPN.^243^ PKC also affects guanosine’s regulatory effects on glial cells, reducing their viability and increasing oxidative damage.^244^ Berberine alleviates diabetes-induced pain by inhibiting transient receptor potential vanilloid 1 (TRPV1) and the PKC pathway, reducing inflammation (Fig. 3).^245^

#### Dyslipidemia

Dyslipidemia significantly contributes to the pathogenesis of DPN, independent of hyperglycemia. Elevated serum triglyceride levels correlate with the progression of DPN and a decline in myelinated fiber density.^52,246^ Rodent models fed high-fat diets develop peripheral neuropathy predominantly affecting small fibers, mirroring human DPN.^247,248^

Saturated fatty acids (SFAs) induce mitochondrial depolarization and reduce the number and velocity of motile mitochondria in DRG neurons.^53^ The detrimental effects are more pronounced with longer-chain SFAs, while monounsaturated fatty acids can mitigate these deficits by enhancing β-oxidation and ATP production.^249,250^ In vivo studies confirm mitochondrial dysfunction in high-fat diet mice, which develop neuropathy marked by reduced nerve conduction velocities and decreased IENFD.^250^ These mice exhibit impaired axonal mitochondrial membrane potential and bioenergetic function, indicating a lack of reserve capacity.^249,251^ Dyslipidemia also affects Schwann cells, leading to mitochondrial dysfunction, endoplasmic reticulum stress, and oxidative stress, ultimately causing cell death.^252^ Oxidized low-density lipoproteins (oxLDL) are injurious to DRG neurons and vascular endothelial cells, contributing to neuronal damage and microvascular dysfunction.^253–255^ OxLDL interacts with various receptors, such as lectin-like oxidized LDL receptor-1 (LOX-1), TLR4, and RAGE, activating inflammatory pathways and increasing ROS accumulation.^256,257^ Gene expression profiling reveals altered lipid metabolism in nerves of patients with DPN and rodent models, suggesting that dysregulated lipid metabolism contributes to DPN.^220,258,259^

While the efficacy of statins in alleviating DPN symptoms remains unclear,^260,261^ emerging lipid-lowering therapies showed potential in improving nerve function in DPN models.^262,263^ Additionally, substituting dietary SFAs with monounsaturated fatty acids improves nerve function in HFD mice without significantly altering plasma metabolic profiles, suggesting that dietary fats influence neuropathy development.^250^ Therefore, targeting dyslipidemia and its downstream effects may offer promising therapeutic avenues for DPN management (Fig. 3).

#### Oxidative stress and mitochondrial dysfunction

Oxidative stress is a critical factor in the development of DPN.^264,265^ Chronic hyperglycemia elevates ROS production through multiple pathways, including enhanced glucose oxidation, activation of NADPH oxidases, and dysfunction of the mitochondrial electron transport chain.^266,267^ Excessive ROS levels lead to oxidative damage to DNA, proteins, and lipids, resulting in neuronal apoptosis and impaired nerve function.^55,268^

High glucose levels impair mitochondrial oxidative phosphorylation, decreasing ATP production and increasing ROS generation.^4^ The resulting energy deficit and accumulation of damaged mitochondria contribute to neuronal degeneration and loss of nerve fibers.^269,270^ An imbalance between mitochondrial biogenesis and fission leads to the formation of small, dysfunctional mitochondria, affecting both neurons and Schwann cells and exacerbating peripheral nerve damage.^271,272^ Additionally, hyperglycemia-induced oxidative and nitrosative stress activates poly(ADP-ribose) polymerase (PARP), a nuclear enzyme involved in DNA repair.^273^ Overactivation of PARP depletes NAD^+^, impairing glycolysis and ATP production, which can lead to neuronal death.^274,275^

Therapeutic strategies targeting oxidative stress and mitochondrial dysfunction include using antioxidants like alpha-lipoic acid (ALA), which improves neuropathic symptoms and nerve conduction velocities in patients with DPN.^276,277^ Mitochondria-targeted antioxidants are being investigated for their ability to protect mitochondrial function and reduce neuronal apoptosis.^278,279^ Lifestyle interventions, particularly aerobic exercise, have also demonstrated benefits in enhancing mitochondrial biogenesis and function, offering a non-pharmacological approach to managing DPN (Fig. 3).^280,281^

#### Inflammation

Inflammation is a pivotal contributor to the pathogenesis of DPN. Chronic hyperglycemia induces a proinflammatory state characterized by elevated levels of cytokines such as tumor necrosis factor-alpha (TNF-α), interleukin(IL)-1 beta, and IL-6.^282^ These cytokines sensitize nociceptive neurons, leading to neuropathic pain, and promote neuronal damage through demyelination and axonal degeneration.^283,284^ Hyperglycemia activates NF-κB signaling pathways, enhancing the expression of inflammatory genes and further amplifying the inflammatory response.^285^ Immune cells infiltrate peripheral nerves in diabetes patients, releasing additional inflammatory mediators that exacerbate nerve injury.^286,287^ PNS-resident macrophages surveil the endoneurial space under normal conditions.^288^ Upon injury, they are joined by infiltrating macrophages to initiate nerve repair.^288,289^ In diabetic conditions, these macrophages can transition to a proinflammatory phenotype, accumulate, and contribute to nerve damage.^290–292^ Accumulating macrophages release cytokines and chemokines, including IL-1β, IL-6, chemokine ligand 2 (CCL2), and TNF-α, intensifying the inflammatory environment and aggravating nerve damage.^293,294^

Depleting macrophages or inhibiting their migration improves mechanical sensation and nerve function in animal models.^295,296^ Cytokine inhibitors and non-steroidal anti-inflammatory drugs are being explored for their potential to alleviate neuropathic pain.^83,284^ Immunomodulatory therapies targeting specific components of the immune response offer promising avenues for reducing nerve inflammation and promoting repair (Fig. 3).^297,298^

#### Microvascular contributions

Microvascular dysfunction plays a significant role in the pathogenesis of DPN. Diabetes-induced endothelial dysfunction impairs blood, causing vasoconstriction.^5,299^ This microvascular impairment reduces oxygen and nutrient delivery to peripheral nerves, leading to nerve ischemia and hypoxia.^300,301^ The hypoxic environment exacerbates oxidative stress, contributing to neuronal degeneration and loss of nerve fibers. Endoneurial microangiopathy, characterized by capillary abnormalities, occurs early in DPN and correlates with clinical, neurophysiological, and morphological abnormalities.^302–304^ Patients with diabetes exhibit increased endoneurial capillary density compared with healthy individuals, suggesting a compensatory response to nerve ischemia.^303^ However, the thickening of the basement membrane correlates with nerve damage, indicating that these vascular changes are detrimental.^302^ Moreover, diabetic nerves demonstrate poor vasodilation of epineurial arterioles, impairing the regulation of endoneurial blood flow.^305,306^ This impaired vasodilatory response appears before decreases in nerve conduction velocity (NCV) and contributes to reduced endoneurial perfusion and ischemia. In experimental models, interventions to improve vascular health enhanced nerve perfusion and promoted nerve regeneration.^307–309^

Endothelial dysfunction is well recognized in DPN.^310^ Systemic markers of endothelial dysfunction and vascular inflammation are associated with DPN.^94,297^ Additionally, hyperglycemia-induced activation of protein PKC affects the expression of VEGF, promotes vasoconstriction, and induces hypoxia.^311^ Diabetes also decreases mediators of blood vessel formation and angiopoietins, contributing to poor nerve health.^312^ Capillary dysfunction with reduced glucose and oxygen extraction has been hypothesized to contribute to endoneurial hypoxia.^313^ Endothelial cells are susceptible to excess nutrients through hyperglycemia- and dyslipidemia-induced oxidative stress, further contributing to nerve damage during diabetes.^225,314^ Administration of VEGF in diabetic models increased NCV and vasa nervorum density, indicating potential therapeutic benefits.^309,315^ Therapies targeting endothelial function are being explored to prevent or mitigate DPN progression (Fig. 3)^316,317^.

#### Impaired insulin signaling

Insulin signaling plays a crucial role in maintaining peripheral nerve health, and its impairment significantly contributes to the pathogenesis of DPN. Insulin receptor signaling stimulates the PI3K/Akt pathway in the PNS, directly related to axon growth, myelin formation, and neuronal survival.^318^ In T2DM, insulin resistance in metabolically active tissues, such as muscle, fat, and liver, leads to hyperglycemia and dyslipidemia.^319,320^ Elevated plasma glucose, HbA1c levels, and abnormal lipid profiles correlate with systemic insulin resistance.^320^ Peripheral nerves develop insulin resistance in T2DM.^16,321,322^ Both neurons and Schwann cells express insulin receptors (IRs), particularly in sensory neurons of DRG and at nodes of Ranvier.^323,324^ Insulin promotes neurite outgrowth, supports neuronal survival,^323,325^ and regulates PNS metabolism.^16,326^ In conditions of nutrient excess, as seen in T2DM, insulin resistance blunts insulin-mediated nutrient and energy responses, impairing nerve metabolism and bioenergetic failure.^327,328^ This disruption contributes to nerve injury by affecting mitochondrial function and axonal trafficking. This mechanism mirrors the distal-to-proximal pattern of nerve damage observed clinically in patients with DPN.^329,330^

Direct insulin signaling in neurons can reverse features of DN independent of glucose levels.^331,332^ Insulin administration near the nerve or skin enhances nerve regeneration and repairs diabetes-induced abnormalities in animal models.^333,334^ However, in patients with T2DM, correcting hyperglycemia with insulin therapy has a limited impact on DPN.^101,335^ This disparity may be attributed to the development of insulin resistance within neurons.^321,336^ Altered phosphorylation of insulin receptor substrate proteins, particularly IRS-1 and IRS-2, is implicated in neuronal insulin resistance,^20,337^ which impairs insulin’s neurotrophic effects, leading to reduced neurite outgrowth and increased vulnerability to injury.^338,339^

In contrast, patients with T1DM often benefit from insulin therapy regarding neuropathic outcomes, as they typically do not exhibit significant insulin resistance.^340^ Understanding the role of insulin signaling and insulin resistance in peripheral nerves is essential for developing targeted therapies for DPN. Recent therapeutic approaches focus on enhancing insulin signaling pathways or reducing insulin resistance within peripheral nerves to improve neuropathic symptoms (Fig. 3).^251^

#### Impaired neurotrophic support

In DPN, levels of neurotrophic factors are significantly reduced due to hyperglycemia-induced metabolic disturbances. The deficiency of neurotrophic support leads to neuronal atrophy, impaired axonal transport, and decreased nerve regeneration capacity.^4,312^ Dysfunctional retrograde transport of neurotrophins from distal axons to neuronal cell bodies contributes to neuronal degeneration. Exogenous administration of neurotrophic factors can promote nerve regeneration and improve functional outcomes in experimental models of DPN.^341^ For instance, treatment with nerve growth factor (NGF) or brain-derived neurotrophic factor (BDNF) enhances neurite outgrowth and supports neuronal survival. However, clinical translation has been challenging due to delivery issues and side effects.^342^ Vitamin B deficiencies, particularly of B12 and B6, can exacerbate neurological dysfunction in DPN.^343^ Supplements like Metanx^®^, which contains L-methylfolate, methylcobalamin, and pyridoxal-5′-phosphate, have improved symptoms by enhancing endothelial function and restoring neurotrophic support.^264,344^ Recent research has explored novel delivery methods to enhance the efficacy and safety of neurotrophic factor-based therapies.^345,346^ For example, gene therapy delivering NGF or its receptors has shown promise in enhancing nerve regeneration in diabetic models.^341^

#### Schwann cell injury

In DPN, hyperglycemia and oxidative stress lead to Schwann cell dysfunction and apoptosis, resulting in demyelination and impaired nerve conduction.^347,348^ High glucose levels inhibit the PI3K/Akt pathway in Schwann cells, leading to overexpression of DNA methyltransferases (DNMT1 and DNMT3a) and upregulation of thioredoxin-interacting protein (TXNIP). This results in decreased autophagy and increased apoptosis, contributing to Schwann cell dysfunction.^349^ Activation of the PI3K/Akt pathway promotes Schwann cell survival and differentiation and enhances myelin formation.^350^ Mitochondrial dysfunction in Schwann cells triggers alterations in lipid metabolism, leading to myelin sheath dysfunction and accumulation of toxic lipid intermediates, which cause axonal degeneration and neuropathy.^351,352^ Lipotoxicity in Schwann cells results in cell dysfunction and death through mechanisms involving endoplasmic reticulum stress, mitochondrial dysfunction, and increased oxidative stress.^252^ Therapeutic strategies to enhance Schwann cell survival and function include using antioxidants, neurotrophic factors, and agents that modulate Schwann cell signaling pathways.^353,354^ For instance, protecting Schwann cells from glucose-induced toxicity improves nerve function and promotes remyelination in experimental models.^355^ Schwann cells provide metabolic support to axons, and disruption of this coupling contributes to DPN.^356,357^ Targeting pathways that improve Schwann cell metabolism and support axonal energy requirements may offer new therapeutic avenues (Fig. 3).

#### CNS involvement

**Fig. 4:**
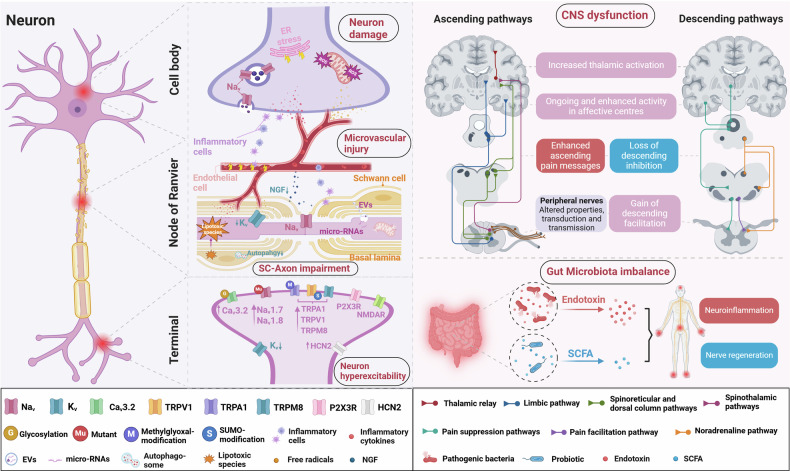
Peripheral and central mechanisms in diabetic neuropathy. Various alterations in peripheral and central neurons contribute to the pathophysiology of DN. In neuronal perikarya, hyperglycemia exacerbates endoplasmic reticulum stress, mitochondrial damage, and oxidative stress, while microvascular changes promote infiltration of inflammatory cells and factors at the neuronal and axonal levels, increasing the expression of voltage-gated sodium channels like Nav1.8, leading to hyperexcitability. Hyperglycemia in nerve fibers disrupts Schwann cell autophagy, increases the release of extracellular vesicles containing microRNAs and lipotoxic species, and reduces nerve growth factor secretion, impairing Schwann-axon interactions and myelin repair. These changes accelerate DN progression and contribute to hyperexcitability in myelinated axons by reducing the expression of shaker-type potassium channels, leading to heightened responses to stimuli and ectopic neuronal activity. Peripheral nerve damage triggers nociceptor hypersensitivity through inflammation, altered transducer activity (e.g., TRPV1, TRPM8, and P2X3R), and ion channel expression changes in sodium, potassium, and calcium channels. Methylglyoxal-induced glycation of nociceptor terminal ion channels forms AGEs, resulting in channel hyperfunction and neuronal hyperexcitability, including increased expression of Nav1.8. In DN, genetic variants (e.g., Nav1.7 and Nav1.8), methylglyoxal-modified TRPA1, SUMO-modified TRPV1, and HCN2 overactivation due to elevated cAMP contribute to neuronal hyperexcitability. Central sensitization results from an imbalance between facilitatory and inhibitory modulation of pain signals in the spinal cord and brain. This involves ascending pathways such as the spinothalamic tract (pain perception), the spinoreticular tract, and pathways through the parabrachial nucleus to the hypothalamus and amygdala, which are associated with autonomic function and emotional responses such as fear and anxiety. Descending pathways can inhibit or facilitate nociceptive signal transmission at the spinal level. The gut microbiota also plays a key role in DN, with dysbiosis linked to inflammation, metabolic disturbances, and nerve damage due to increased intestinal permeability and endotoxin translocation. Beneficial metabolites such as SCFAs, including butyrate, support nerve regeneration and reduce neuroinflammation, offering new therapeutic opportunities for DN management. AGEs advanced glycation end products, cAMP cyclic adenosine monophosphate, CNS central nervous system, DN diabetic neuropathy, DPN diabetic peripheral neuropathy, EVs extracellular vesicles, HCN2 hyperpolarization-activated cyclic nucleotide-gated 2, P2X3R P2X receptor subtype 3, SC Schwann cell, SCFAs short-chain fatty acids, TRPV1 transient receptor potential vanilloid 1, TRPA1 transient receptor potential ankyrin 1, TRPM8 transient receptor potential melastatin 8

The CNS plays a significant role in the development and maintenance of DPN. Hyperglycemia-induced changes affect the spinal cord and brain regions involved in pain processin.^358,359^ Structural changes, such as spinal cord atrophy and reduced gray matter volume in the primary somatosensory cortex, have been observed in patients with DPN.^359,360^ Functional abnormalities also play a critical role in DPN, including altered thalamic activity and disrupted thalamocortical connectivity. These changes lead to central sensitization, where non-painful stimuli are perceived as painful (allodynia), and pain responses are amplified (hyperalgesia).^361–363^ Proton magnetic resonance spectroscopy studies have shown a reduced N-acetylaspartate to creatine ratio in the thalamus and parietal white matter pathways of DPN patients, indicating neuronal dysfunction.^364,365^ PDPN is associated with increased thalamic vascularity and functional reorganization of the primary somatosensory cortex.^362,366^ Multimodal therapeutic approaches combining pharmacotherapy, CBTs, and neuromodulation techniques are being explored to alleviate neuropathic pain and improve patient outcomes.^84,367^ The involvement of the CNS opens new avenues for research and the development of novel therapeutic targets aimed at modulating central pain pathways in DPN (Fig. 4).

#### Cellular autophagy and cell death

In DPN, hyperglycemia impairs autophagy by inhibiting key autophagy-related proteins, resulting in the accumulation of dysfunctional mitochondria and protein aggregates.^368,369^ Enhancing autophagy can mitigate nerve damage in DPN. Rapamycin, which activates autophagy by inhibiting the mTOR pathway, has shown neuroprotective effects in experimental models.^370,371^ Ongoing research aims to identify safe and effective autophagy modulators as potential therapeutic strategies for DPN.^372,373^ The mTOR pathway plays a significant role in DPN development by affecting autophagy and apoptosis in Schwann cells, myelin formation, and lipid metabolism, thereby influencing myelin and axonal integrity.^242^ Astragaloside IV inhibits the PI3K/Akt/mTOR pathway by upregulating miR-155 expression, thereby preventing apoptosis and enhancing autophagy to alleviate myelin damage in Schwann cells and improve neurological function in DPN rat models.^374^ Similarly, Lycium barbarum polysaccharides exert a protective effect by inhibiting the mTOR/p70S6K pathway, promoting autophagy in Schwann cells.^375^ Therefore, the state of the mTOR pathway affects the balance between autophagy and apoptosis in Schwann cells.

Hyperglycemia-induced oxidative stress and inflammation activate intrinsic and extrinsic apoptotic pathways, leading to programmed cell death of neurons and Schwann cells.^376,377^ Necroptosis has been implicated in the pathogenesis of DN.^378^ Agents that inhibit apoptotic signaling, such as caspase and necroptosis inhibitors, are currently being explored for their potential neuroprotective effects. Excessive pyroptosis leads to rapid rupture of the plasma membrane and the excessive release of proinflammatory cytokines and chemokines, which results in neuroinflammation that triggers a cascade of secondary injuries culminating in neuronal death.^379–381^ Glucagon-like peptide-1 receptor agonists (GLP-1RAs) may act directly on DRG neurons by inhibiting the activation of inflammatory vesicle signaling pathways.^382,383^ An in-depth understanding of the underlying mechanisms of cellular pyroptosis is essential to determine its role in the pathogenesis of DPN and develop effective therapeutic agents targeting the pyroptosis signaling pathway to alleviate neuropathic symptoms.

#### Genetic and epigenetic factors

Genetic predispositions significantly influence the risk of developing DPN. Variations in genes associated with inflammation, oxidative stress response, and neuronal function have been linked to increased susceptibility to neuropathy in diabetes patients.^384,385^ Additionally, GWAS has identified several novel genetic loci associated with DPN, underscoring the complex genetic architecture underlying this condition.^78,386^ Epigenetic modifications alter gene expression without changing the DNA sequence and can be influenced by environmental factors, including hyperglycemia.^387,388^ These epigenetic changes contribute to the activation of pathogenic pathways involved in DPN.^389,390^ Recent research has identified specific genetic and epigenetic markers for early detection and risk stratification and developed epigenetic therapies to reverse harmful modifications and restore normal gene expression.^242,374^ Therapeutic approaches targeting epigenetic regulators are being explored to mitigate the progression of DPN.^389,390^ Moreover, improvements in CRISPR-Cas9 technology present opportunities for accurate genetic and epigenetic alterations to rectify inherited genetic variants and epigenetic markers associated with DPN.^391,392^ Understanding the interplay between genetic and epigenetic factors is crucial for developing personalized medicine strategies to effectively prevent and treat DPN.

#### Non-coding RNAs

Non-coding RNAs, including microRNAs (miRNAs), long non-coding RNAs (lncRNAs), and circular RNAs (circRNAs), are crucial regulators in the pathophysiology of DPN. These molecules modulate gene expression at transcriptional and post-transcriptional levels, influencing inflammation, oxidative stress, and neuronal repair.^393,394^ For instance, miR-146a regulates inflammatory responses by targeting IRAK1 and TRAF6, and its decreased expression contributes to increased inflammation in DPN.^395^ Similarly, lncRNAs can act as competing endogenous RNAs (ceRNAs), sequestering miRNAs and affecting their regulatory functions.^396^ Circulating non-coding RNAs are potential biomarkers for early diagnosis and monitoring of disease progression.^397,398^

Moreover, Schwann cell-derived extracellular vesicles (EVs) play a significant role in DPN pathogenesis. Hyperglycemia-treated Schwann cells release pathologic EVs that suppress axonal growth in vitro and accelerate DPN development in vivo in db/db T2D mice.^399^ These EVs contain elevated levels of miR-28, miR-31a, and miR-130a, which reduce the expression of target proteins.^399^ In contrast, homeostatic Schwann cell-derived EVs increase DRG neurite outgrowth in vitro and restore small and large fiber functions upon injection into db/db mice.^400^ The underlying neuroprotective mechanisms involve increased levels of miR-21, miR-27a, and miR-146a, along with decreased expression of target proteins.^401,402^ miR-21 in Schwann cell-derived EVs has beneficial effects on neurite outgrowth in vitro.^401^ These findings suggest that Schwann cells under impaired metabolic conditions release pathologic EVs that promote nerve degeneration and DPN, whereas homeostatic EVs may facilitate nerve repair.

#### Gut microbiota

The gut-brain axis is a pivotal area of interest in studying DPN. Dysbiosis is associated with systemic inflammation and metabolic dysfunction in diabetes, both of which can exacerbate DPN.^403,404^ Alterations in gut microbiota can increase intestinal permeability, leading to the translocation of endotoxins and the activation of immune responses that contribute to peripheral nerve damage.^405^ Modulating the gut microbiota through dietary interventions, probiotics, and fecal microbiota transplantation can positively influence neuronal health and alleviate neuropathic symptoms.^406–408^ Microbial metabolites, such as short-chain fatty acids (SCFAs), play a significant role in neuroinflammation and nerve regeneration.^409,410^ This holds promise for developing novel therapeutic strategies targeting the gut microbiota to manage DPN effectively.^388,389^ Butyrate enhances the integrity of the blood-nerve barrier and reduces inflammatory cytokine production, protecting peripheral nerves from damage.^411^ Additionally, specific probiotic strains have been identified that can restore gut microbiota balance, reduce systemic inflammation, and improve nerve conduction in diabetic models.^412,413^ Fecal microbiota transplantation (FMT) has also been explored as a method to reset gut microbiota composition, demonstrating improvements in metabolic parameters and neuropathic pain in preclinical studies.^414,415^ Metabolites produced by gut bacteria influence host energy metabolism, immune responses, and neuronal function.^416^ Understanding these interactions is crucial for identifying biomarkers for early detection and for developing targeted microbiota-based therapies.^297,417^ Ongoing clinical trials are assessing the efficacy of microbiota modulation in patients with DPN to translate these findings into clinical application.^418,419^

Although research on the pathophysiological mechanisms of DN covers multiple aspects, several pathways remain poorly understood, and the clinical application of treatment strategies remains limited. Future should aim to investigate these complex mechanisms, develop precise therapeutic methods, and advance personalized medicine and emerging fields, such as gut microbiota research to enhance treatment outcomes.

### Mechanisms of PDPN

Among patients with DN, 30–50% develop neuropathic pain.^38^ This pain commonly presents as spontaneous burning sensations in the feet, and patients may also report positive sensory symptoms such as brush-evoked allodynia (pain from normally non-painful stimuli) and paresthesias, which are often accompanied by sensory loss, leading patients to describe the paradox of their feet being continuously painful yet insensate to touch.^420^ Neuropathic pain arises due to persistent maladaptive structural and functional changes within the somatosensory system following peripheral nerve injury.^421^ Research, primarily using experimental models of mechanical nerve damage, has explored the mechanisms underlying neuropathic pain, revealing increased excitation and facilitation of pain signals, along with a loss of inhibition in both the PNS and the CNS.^421^ However, neurophysiological measurements, molecular pathways, and pathological findings do not fully explain the presence of neuropathic pain in diabetes.^421–423^ Structural and functional changes within the somatosensory system, along with maladaptive responses in the CNS and PNS, contribute to the mechanisms underlying PDPN (Fig. 4).^422,423^

#### Peripheral mechanisms of PDPN

Peripheral mechanisms in PDPN involve multiple factors. Small fibers are preferentially impaired in PDPN. However, measurements of IENFD and basic QST have not conclusively demonstrated their preferential involvement in PDPN.^422^ Two large cross-sectional studies using detailed QST profiling found greater thermal insensitivity in painful than painless DPN, suggesting more advanced small fiber dysfunction.^69,70^ Small nerve fibers may become sensitized following increased distal terminal injury and regeneration in the presence of neurotrophins.^87^ Alternatively, hyperexcitable small fiber neurons in PDPN might be more susceptible to bioenergetic failure, increasing distal nerve fiber degeneration.^424^ Compared with painless DPN, PDPN is associated with more severe corneal small fiber damage^425–427^ and increased markers of degeneration (axonal swelling) and regeneration.^428,429^ However, the reported findings are inconsistent, possibly due to selection bias and small study sizes.^421,425,430^

Studies have demonstrated greater cardiac autonomic dysfunction in PDPN than in painless DPN,^421,427,431^ while others found no association.^432,433^ Profound abnormalities in the arteriovenous anatomy have been described on the epineurial surface in patients with acute painful neuropathy during rapid glycemic control.^434^ Patients with PDPN have increased epineurial blood flow and intravascular oxygen saturation, perhaps secondary to arteriovenous shunting due to sympathetic denervation.^84^ The topical application of vasodilators provides pain relief in PDPN, suggesting a role for hypoxia and/or vascular dysfunction in the generation of neuropathic pain in DPN.^435,436^

Neuronal injury promotes a neuroinflammatory response, leading to the accumulation of immune cells and the release of inflammatory cytokines, contributing to neuronal hypersensitivity. In PDPN, these mechanisms facilitate ectopic neuronal activity and enhance response to sensory stimuli.^69^ Cross-sectional studies have identified inflammatory mediators associated with PDPN.^437,438^ Elevated serum levels of soluble intracellular adhesion molecule 1 (sICAM-1), indicative of increased vascular inflammation and/or endothelial dysfunction, have also been reported in PDPN.^432,433^ Microglia may contribute to neuropathic pain through impaired descending modulation, altering the balance between descending inhibition and excitation toward the latter.^432^ Minocycline, known to reduce microglial activation, reduces neuropathic pain in Sprague–Dawley rat models of T1DM with DPN.^433^

Methylglyoxal, a reactive dicarbonyl by-product of glycolysis, induces post-translational modifications of the voltage-gated sodium channel Nav1.8, increasing the excitability of nociceptive neurons.^434^ Despite showing promise as a biomarker of neuropathic pain, a cross-sectional study found no association between methylglyoxal and PDPN.^428^ Peripheral nociceptor terminals respond to noxious stimuli via an array of receptors and ion channels to initiate an action potential. The altered expression, modification, and function of these transducers as TRPV1, transient receptor potential ankyrin 1 (TRPA1), and transient receptor potential melastatin 8 (TRPM8), contribute to peripheral sensitization and may play an important role in pain phenotyping.^11,84,439–446^ Painful neuropathies cause alterations in ion channels.^435^ Gain of function of sodium channels leads to increased neuronal excitability, signal transduction, and neurotransmitter release.^436^ Preclinical^69,434^ and human genetic studies^69,437,438^ in PDPN have shown evidence of enhanced function of voltage-gated sodium channels. Concurrently, there is a downregulation in the expression of components of voltage-gated potassium channels, which normally regulate neuronal excitability, repolarization, and frequency of action potentials.^438^ Voltage-gated calcium channels (VGCCs) are functionally diverse and involved in peripheral and central nociception.^437^ T-type (CaV3.2) calcium channels are especially significant in DN.^447–450^ VGCCs, present in the presynaptic terminal, are responsible for neurotransmitter release and the propagation of pain signals to second-order neurons.^438^ Components of subtypes of these channels are upregulated in rodent DRG cells displaying neuropathic pain; agents targeting these channels provide analgesia.^1,11^ Ligand (ATP)-gated non-selective cation channels are found on neurons, Schwann cells, and microglia.^451^ In streptozotocin (STZ)-induced diabetic rat and mouse models, elevated expression of P2X2 and P2X3 receptors in the DRG, along with increased current density and P2X4 receptor expression on satellite glial cells, indicates that P2X4 receptor activation in the DRG significantly contributes to DPN-induced mechanical pain hypersensitivity (Fig. 4)^452^.

#### Dysfunction within the CNS

Brain imaging studies have demonstrated an association between PDPN and structural changes in key somatosensory regions, such as the thalamus and somatosensory cortex, and altered functional connectivity within ascending pain pathways and cortical regions involved in pain processing. Disruptions in the descending pain modulatory system can amplify or inhibit pain signals.^366,453,454^ The relationship between these structural and functional brain changes and metabolic factors remains poorly understood, with potential mechanisms including vascular injury, oxidative stress, osmotic imbalances, and glial and neuronal damage.^455^ Future research should elucidate how aberrant sensory afferent activity interacts with maladaptive CNS plasticity to initiate and sustain neuropathic pain.^456^

In the spinal cord, the influx of neuropathic signals to the dorsal horn leads to central sensitization, characterized by increased spontaneous neuronal activity, lowered activation thresholds for peripheral stimuli, heightened responses to sub-threshold stimuli, and expansion of receptive fields.^457^ Activation of N-methyl-D-aspartate (NMDA) receptors amplifies and prolongs spinal dorsal horn neuron responses, further contributing to pain perception.^421,458^ Alterations in the balance of descending modulatory pathways are hypothesized to sustain chronic pain states, with a reduction in inhibitory control and an increase in facilitatory activity within both the brain and spinal cord.^421^ Specifically, dysfunction within the ventrolateral periaqueductal gray (vlPAG)-mediated descending pain modulatory system has been reported in patients with PDPN.^454^ Moreover, centrally mediated factors, such as cognitive processes (e.g., attention, distraction, catastrophizing), contextual beliefs about pain, and emotional states (e.g., depression, anxiety), significantly influence the pain experience.^421,459,460^

The thalamus plays a pivotal role in modulating and processing somatosensory inputs before they reach the cerebral cortex and may serve as a central biomarker for neuropathic pain in diabetes mellitus.^459^ In rat models of T1DM, thalamic ventroposterolateral neurons exhibit hyperexcitability in response to stimuli.^461^ These neurons also demonstrate increased spontaneous activity independent of ascending afferent input and possess enlarged receptive fields, suggesting that thalamic neurons function as central generators or amplifiers of pain in DPN.^462^ Magnetic resonance perfusion imaging studies have identified increased thalamic vascularity in patients with PDPN during resting states, likely reflecting heightened neuronal activity.^463,464^ Additionally, decreased thalamocortical functional connectivity observed through resting-state functional MRI correlates with PDPN.^465^

The anterior cingulate cortex, essential for emotional pain processing, shows increased blood flow in patients with PDPN, a hyperperfusion state that normalizes following duloxetine treatment.^438^ Functional MRI studies have reported augmented blood oxygen level-dependent (BOLD) responses in limbic and striatal structures in response to heat pain in PDPN patients.^437^ Furthermore, structural and functional alterations in the primary SSC are associated with pain phenotypes in DPN.^432,438^ Notably, the analgesic response to intravenous lidocaine, a sodium channel blocker, in PDPN patients correlates with increased SSC volume and enhanced functional connectivity between insular and corticolimbic circuits.^432^ These findings highlight the necessity for advanced imaging studies to further clarify the relationship between CNS alterations and individualized pain phenotypes in PDPN (Fig. 4). Current research on the mechanisms underlying PDPN has identified multiple peripheral and central factors; however, the specific interactions between these mechanisms remain nebulous, and existing models cannot fully explain the presence of diabetic neuropathic pain. Future studies should focus on elucidating the complex interactions within the somatosensory system and developing targeted therapies based on advanced imaging and molecular insights.

### DAN

DAN is a serious and common complication of diabetes mellitus that affects the ANS, which controls involuntary bodily functions. DAN can impact various organ systems, including the cardiovascular, gastrointestinal, genitourinary, and sudomotor systems, leading to significant morbidity and mortality among diabetes patients.

#### CAN

CAN is characterized by impaired autonomic control over cardiovascular function, resulting from damage to the sympathetic and parasympathetic fibers that innervate the heart and blood vessels. This imbalance between sympathetic and parasympathetic activity leads to resting tachycardia, reduced HRV, orthostatic hypotension, and an increased risk of silent myocardial ischemia and sudden cardiac death.^5,466^ Microvascular insufficiency leads to ischemia of autonomic nerves. Inflammation and autoimmune mechanisms also play significant roles in the pathogenesis of CAN.^112,467^ Recent studies have focused on the early detection of CAN using advanced diagnostic tools like spectral analysis of HRV and cardiac imaging techniques. There is growing interest in the role of inflammatory markers and genetic predispositions in the development of CAN.^468,469^ Moreover, therapeutic interventions targeting metabolic pathways, oxidative stress, and inflammation are being explored to prevent or slow the progression of CAN.^42^

#### Gastrointestinal autonomic neuropathy

Gastrointestinal autonomic neuropathy affects the enteric nervous system, leading to dysregulation of gastrointestinal motility and function. This dysregulation can manifest as esophageal dysmotility, gastroparesis (delayed gastric emptying), intestinal enteropathy, constipation, or diarrhea.^470,471^ Impaired vagal nerve function exacerbates gastrointestinal symptoms.^472,473^ Recent advancements in imaging and motility testing have enhanced the early diagnosis of GAN, utilizing tools such as high-resolution manometry and wireless motility capsules. Therapeutic strategies include prokinetic agents, dietary modifications, and interventions targeting the gut microbiota, aiming to restore normal gastrointestinal function and alleviate symptoms.^474^

#### Genitourinary dysfunction

Genitourinary dysfunction in DAN encompasses both bladder dysfunction (diabetic cystopathy) and sexual dysfunction. In males, ED is a prevalent complication, while females may experience sexual dysfunction, including decreased libido and arousal disorders.^475,476^ Chronic hyperglycemia-induced neuronal damage to the autonomic fibers innervating the bladder and genitalia leads to impaired neurotransmission.^477^ Additionally, vascular changes, hormonal imbalances, and psychological factors contribute to genitourinary dysfunction in diabetes.^478^ Addressing modifiable risk factors and providing psychosocial support enhance patient outcomes.^479,480^ These advancements offer promising avenues for managing genitourinary complications in DN.^481^ Emerging evidence indicates that the gut microbiota plays a significant role in the development of DAN. Dysbiosis, or an imbalance in gut microbial composition, can influence the ANS through metabolic, immune, and neural pathways. Microbial metabolites, such as SCFAs and lipopolysaccharides (LPS), may affect gut permeability, systemic inflammation, and neural function.^482^ Recent studies have demonstrated alterations in the gut microbiota of patients with diabetic CAN.^483^ Modulating the gut microbiota through probiotics, prebiotics, and dietary interventions has shown the potential to improve autonomic function and reduce inflammation.^41^ Understanding the gut-brain axis in the context of DAN opens new avenues for therapeutic interventions.

Current research on DAN has advanced diagnostic tools and treatment strategies, but the understanding of its complex mechanisms and the impact of gut microbiota remains insufficient. Future studies should delve deeper into these mechanisms, develop more effective diagnostic methods, and create personalized treatment plans to improve patient outcomes.

### Focal and multifocal neuropathies

The primary pathophysiological mechanism underlying focal and multifocal neuropathies is ischemic injury resulting from the occlusion or compromise of the vasa nervorum—the small blood vessels supplying peripheral nerves. Diabetes-induced microvascular alterations, including the thickening of the capillary basement membrane and endothelial hyperplasia, diminish blood flow to nerves, leading to ischemia and subsequent nerve fiber damage.^484^ Recent research has highlighted the role of immune dysregulation in diabetic focal neuropathies, with nerve biopsies revealing inflammatory infiltrates that suggest potential benefits from immunosuppressive therapies.^171,485^ Advances in imaging techniques have enhanced the diagnosis and monitoring of these neuropathies, allowing for earlier and more accurate identification of affected nerves.^486^ Clinically, early recognition and differentiation of focal and multifocal neuropathies are crucial for effective management, which includes stringent glycemic control, pain management, and addressing underlying vascular or inflammatory processes. In cases where inflammation is prominent, immunosuppressive therapies may be warranted to prevent further nerve damage and improve patient outcomes.^187^ Ongoing research continues to explore the efficacy of novel therapeutic interventions and the optimization of existing treatments to better manage these complex neuropathic conditions.

## Current therapies for DN

### Targeted prevention and treatment of DN

**Fig. 5:**
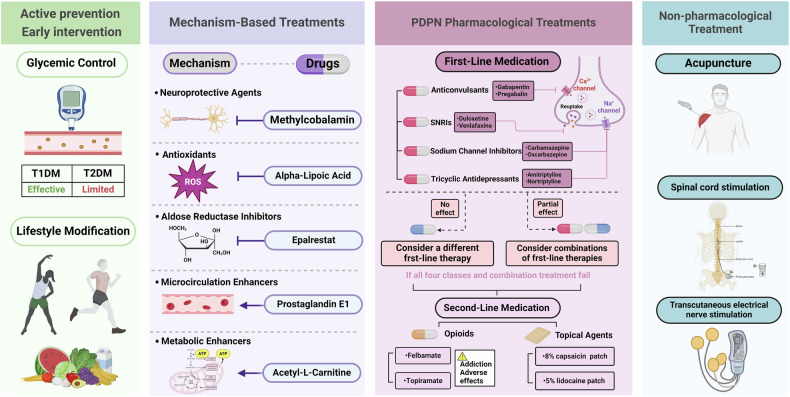
Management strategies for diabetic neuropathy. Maintaining good glycemic control helps prevent the development of neuropathy in patients with type 1 diabetes, though the effect is less pronounced in those with type 2 diabetes. Lifestyle modifications, such as a balanced diet and regular exercise, are recommended for all patients. Mechanism-based treatments focus on addressing the underlying pathophysiology of diabetic neuropathy to alleviate symptoms and slow disease progression. Current therapeutic strategies include neuroprotective agents (methylcobalamin), antioxidants (alpha-lipoic acid), aldose reductase inhibitors (epalrestat), microcirculation enhancers (prostaglandin E1), and metabolic enhancers (acetyl-L-carnitine). PDPN is a frequent and debilitating complication that severely impacts patients’ quality of life. First-line and second-line treatments for PDPN involve various drug classes, including anticonvulsants (pregabalin and gabapentin), serotonin-norepinephrine reuptake inhibitors (duloxetine and venlafaxine), sodium channel inhibitors (carbamazepine and oxcarbazepine), and tricyclic antidepressants (amitriptyline and nortriptyline). Opioids (tapentadol and tramadol) should be avoided due to their significant adverse effects and high potential for addiction. Non-pharmacological therapies, such as TENS, SCS, and acupuncture, are personalized based on individual patient needs. TENS transcutaneous electrical nerve stimulation, SCS spinal cord stimulation, PDPN painful diabetic peripheral neuropathy

DN is a common and serious complication of diabetes mellitus, leading to significant morbidity and a diminished quality of life. Despite its prevalence, there are currently no specific therapeutic strategies that can reverse diabetic nerve damage once it has occurred. Early signs of DN are often subtle and nonspecific, which can lead to delayed diagnosis and progression to irreversible stages of nerve damage.^101^ Therefore, proactive prevention and early intervention are crucial to mitigate the impact of this condition (Fig. 5).

#### Glycemic control

Intensive glycemic control is vital in reducing the incidence and progression of DN in patients with T1DM. Maintaining blood glucose levels as close to normal as possible significantly reduced the risk of developing DPN and DAN.^487,488^ Long-term follow-up studies from the Epidemiology of Diabetes Interventions and Complications (EDIC) research have confirmed these benefits.^489^ In T2DM, the role of glycemic control in preventing neuropathy is more complex. The Action to Control Cardiovascular Risk in Diabetes trial and the Veterans Affairs Diabetes Trial have shown that while intensive glycemic control may slow the progression of neuropathy, it does not significantly prevent its onset.^490,491^ The UK Prospective Diabetes Study indicated a modest reduction in neuropathy risk with improved glycemic control.^492^ These findings suggest that hyperglycemia is only one of multiple factors contributing to neuropathy in T2DM, highlighting the need for a multifactorial approach to management. While aiming for optimal glycemic targets, it is essential to balance the benefits of tight glucose control against the risks of hypoglycemia, especially in individuals with long-standing diabetes or comorbidities.^493^ Personalized glycemic targets should be established based on patient-specific factors, including duration of diabetes, age, comorbid conditions, and risk of hypoglycemia.^494^

#### Lifestyle modification

Lifestyle modification plays a pivotal role in the prevention and management of DN. Risk factors, such as obesity, hypertension, dyslipidemia, smoking, and physical inactivity, are associated with an increased incidence and severity of neuropathy.^299,495^ Adopting a healthy diet that emphasizes whole grains, fruits, vegetables, lean proteins, and healthy fats can improve glycemic control and reduce cardiovascular risk factors.^496^ Specific dietary patterns, such as the Mediterranean diet, have been associated with reduced inflammation and improved metabolic profiles in diabetes patients.^497^ Regular physical activity has been shown to improve insulin sensitivity, aid in weight management, and enhance cardiovascular health. Exercise may also have direct neuroprotective effects by promoting nerve regeneration and improving NCV.^498,499^ Smoking is an independent risk factor for DN and microvascular complications.^500^ Smoking cessation is strongly recommended to reduce the risk of neuropathy progression and improve overall health outcomes. Health education is vital for patients to make informed lifestyle choices. Structured education programs can improve self-management skills, adherence to treatment plans, and awareness of the importance of regular monitoring for early signs of neuropathy.^501,502^

**Key recommendations:**

- **T1DM patients:** Early and intensive glycemic control is recommended to prevent or delay the onset and progression of DPN and DAN.
- **T2DM patients:** Glycemic control is recommended to slow the progression of neuropathy; however, its effect on prevention is less pronounced than T1DM.
- **Individualized targets:** Glycemic targets should be individualized to balance efficacy and safety, particularly in patients with advanced disease or multiple comorbidities.
- **Lifestyle interventions:** Implement lifestyle modifications, including a healthy diet, regular physical activity, and smoking cessation, to reduce the risk of neuropathy in patients with prediabetes, metabolic syndrome, and T2DM.
- **Patient-centered approach:** Provide individualized education and support to encourage adherence to lifestyle changes and self-care behaviors.

### Mechanism-based treatments

Mechanism-based treatments aim to target the underlying pathophysiological processes of DN to alleviate symptoms and prevent disease progression. Current therapies encompass various strategies, including neuroprotective agents, antioxidants, ARIs, microcirculation enhancers, metabolic enhancers, and certain traditional herbal medicines (Fig. 5).

#### Neuroprotective agents

Methylcobalamin, an active form of vitamin B12, plays a crucial role in nerve regeneration and myelin sheath formation and promotes the synthesis of nucleic acids and proteins within nerve cells, facilitating axonal regeneration and improving NCV. Recent clinical trials have demonstrated that methylcobalamin can significantly improve neuropathic symptoms and nerve function in patients with DPN.^503,504^

#### Antioxidants

ALA is a potent antioxidant that mitigates oxidative stress, a key contributor to DN, inhibits lipid peroxidation, improves endoneurial blood flow, and enhances nerve glucose uptake and energy metabolism. Clinical studies have shown that ALA significantly improves neuropathic symptoms and nerve conduction velocities.^505–507^

#### ARIs

Epalrestat is an aldose reductase inhibitor that prevents the accumulation of sorbitol and fructose within nerve cells, which can cause osmotic stress and neuronal damage. By inhibiting this pathway, epalrestat improves metabolic imbalances associated with DN. Epalrestat also alleviates subjective symptoms and enhances NCV.^197,508^

#### Microcirculation enhancers

Prostaglandin E1 (PGE1) and its analogs act as vasodilators, improving microcirculation by relaxing the vascular smooth muscle and reducing platelet aggregation. PGE1 enhances symptoms and nerve conduction velocities in DPN patients, particularly when combined with neuroprotective agents, such as methylcobalamin.^509,510^ Additionally, Pentoxifylline improves microcirculation by decreasing blood viscosity and enhancing erythrocyte flexibility, which improves NCV and reduces neuropathic symptoms in DPN patients.^309^ Kallidinogenase, another microcirculation enhancer, increases capillary blood flow by generating bradykinin, leading to vasodilation and enhanced tissue perfusion. Clinical studies indicate positive effects on DPN symptoms and nerve function.^511^

#### Metabolic enhancers

Acetyl-L-carnitine (ALC) facilitates fatty acid transport into mitochondria, enhancing cellular energy production, and has neuroprotective properties that alleviate neuropathic pain, improve sensory function, and enhance nerve conduction.^512,513^ ALC significantly improved pain reduction and nerve fiber regeneration in DPN patients treated with ALC.^512,513^

#### Herbal medicines

Traditional Chinese medicine includes various herbal preparations being explored for their potential benefits in managing DN. Mudan Granules, containing herbs such as *Salvia miltiorrhiza* and *Angelica sinensis*, are believed to promote blood circulation and relieve pain. Mudan granules may have comparable efficacy to methylcobalamin in treating DPN, improving symptoms and nerve conduction velocities.^514^ Dan Shen dripping pills, derived from *Salvia miltiorrhiza*, improve microcirculation, inhibit platelet aggregation, and reduce oxidative stress. These pills can enhance symptoms and nerve conduction velocities in DPN patients, both alone and in combination with methylcobalamin.^515,516^

Current treatments for DN focus on glycemic control, lifestyle modifications, and mechanism-based therapies but not on reversing nerve damage. Some therapies have limited efficacy. In the future, it is necessary to develop more effective reversal therapies, optimize early diagnostic methods, and advance personalized treatment strategies to improve patient outcomes and quality of life.

### Treatment of PDNP

Effective management of PDPN primarily relies on pharmacological interventions, supplemented by non-pharmacological therapies tailored to individual patient needs.

#### Pharmacological treatments

Recent guidelines recommend the following classes of medications for the treatment of PDPN (Fig. 5):

**First-line medications**

**Anticonvulsants**

- **Pregabalin:** Demonstrates significant pain reduction and improves sleep and quality of life in several randomized controlled trials (RCTs).^17,517^ Pregabalin has linear pharmacokinetics, allowing for twice-daily dosing and a faster onset of action.
- **Gabapentin:** Effective in reducing neuropathic pain symptoms but requires gradual titration and thrice-daily dosing due to its pharmacokinetic profile.^17,518^ However, gabapentin is less expensive, which is an important consideration for patients, especially those with high-deductible health plans in the United States.^519^

**Serotonin-norepinephrine reuptake inhibitors (SNRIs)**

- **Duloxetine:** The most prescribed SNRI for PDPN and has regulatory approval for treating neuropathic pain.^367,520^ Duloxetine relieves pain by increasing the synaptic availability of serotonin and norepinephrine in descending pathways, enhancing endogenous pain modulation. Duloxetine significantly reduces pain scores and improves functional outcomes.^521–524^ The most common adverse effects are nausea, dry mouth, dizziness, somnolence, fatigue, and constipation.
- **Venlafaxine:** An alternative SNRI that has demonstrated efficacy in treating PDPN in several studies.^525,526^ However, in a randomized controlled trial involving 244 patients with PDPN, 7 treated with venlafaxine developed clinically significant electrocardiogram changes during treatment, making it less preferred in patients with cardiovascular conditions.^525^

**Sodium channel inhibitors**

- **Carbamazepine and oxcarbazepine:** Recently included as first-line treatments in guidelines, carbamazepine and oxcarbazepine reduce neuronal excitability by blocking voltage-gated sodium channels.^37^ They are used when α_2_δ ligands and SNRIs are ineffective or contraindicated.
**Tricyclic antidepressants (TCAs)**
- **Amitriptyline and nortriptyline:** Effective in managing PDPN by inhibiting monoamine reuptake and blocking sodium channels.^421,527^ Amitriptyline is the most frequently prescribed TCA and is relatively inexpensive.^23^ However, their use is limited by anticholinergic side effects, especially in elderly patients and those with cardiovascular disease.^527,528^

**Second-line medications**

**Opioids and atypical opioids**

- **Tapentadol:** A centrally acting analgesic with both opioid agonist and norepinephrine reuptake inhibition properties. It may be considered in refractory cases but carries risks of dependence and adverse effects.^529^
- **Tramadol:** should be used cautiously due to similar concerns regarding dependence and side effects.^530^ While opioids can provide pain relief, their high risk of addiction and other safety concerns limit their use. The most recent ADA position statement does not recommend opioid use as first- or second-line therapies for treating neuropathic pain associated with DN.^531,532^ When opioids are prescribed, very close monitoring of the patient is advised.^533^

Topical agents such as the capsaicin 8% patch provide localized pain relief by desensitizing nociceptive fibers, while the lidocaine patch 5% is useful for focal neuropathic pain with a favorable safety profile.^534,535^ Combination therapy, starting with a single agent and titrating to the maximum tolerated dose, may enhance efficacy when pain control is inadequate, with recent studies suggesting superior pain relief compared to monotherapy.^520,536^

#### Non-pharmacological treatments

Non-pharmacological treatments encompass acupuncture, which has gained acceptance as a complementary therapy for PDPN by modulating pain through endogenous opioid release and altering neurotransmitter levels, significantly reducing pain intensity.^537,538^ Electrical stimulation therapies, including transcutaneous electrical nerve stimulation (TENS) and spinal cord stimulation (SCS), have demonstrated effectiveness in reducing neuropathic pain intensity and improving quality of life.^539,540^ Lifestyle interventions, such as a healthy diet, regular physical activity, and smoking cessation, are essential in managing PDPN by improving glycemic control, enhancing nerve function, and reducing pain perception.^541,542^ Physical therapy and rehabilitation, including balance training, gait training, and strength exercises, improve functional mobility and reduce the risk of falls.^22^ Additionally, CBT addresses the psychological aspects of chronic pain, helping patients to cope with and manage pain, thereby enhancing treatment outcomes when combined with pharmacotherapy (Fig. 5)^22^. Current treatments for PDPN primarily rely on pharmacological and non-pharmacological interventions; however, existing medications have limited efficacy and potential side effects, and evidence for non-pharmacological therapies remains limited. Future studies should focus on developing more effective and safer treatments and optimizing multimodal therapeutic strategies to improve pain management and patient quality of life.

### Management of DPN-related complications

Effective management of complications arising from DPN is essential to reduce morbidity and improve patient outcomes. Foot complications are a significant concern; therefore, regular foot examinations are recommended for patients with diabetes at least annually to identify risk factors for ulceration and amputation.^543^ Additionally, patient education on proper foot care, including daily inspections, appropriate footwear, and maintaining foot hygiene, is crucial in preventing severe foot-related complications.^544^ Interventions, such as pressure offloading using therapeutic footwear and insoles, help redistribute pressure and prevent ulcer formation, while establishing multidisciplinary foot care teams involving endocrinologists, podiatrists, surgeons, and nurses reduces amputation rates and healthcare costs.^545,546^ Patients with DPN are also at an increased risk of falls due to sensory loss, balance impairment, and muscle weakness. Fall prevention strategies are, therefore, vital. Assessment of fall risk through evaluating gait and balance can identify patients who are at high risk.^547^ Interventions, such as balance and strength training, improve proprioception and reduce the risk of falls by enhancing muscle strength and coordination.^548^ Additionally, a medication review to adjust or discontinue medications that may impair cognition or balance is recommended to decrease fall risk.^549^ Chronic pain and disability associated with DPN can lead to significant psychological distress. Psychological support is, therefore, an integral component of comprehensive DPN management. Assessment using validated scales can identify patients in need of mental health support.^550^ CBT and mindfulness-based stress reduction have been shown to improve coping strategies and reduce pain perception, thereby enhancing overall treatment outcomes.^551^ Additionally, support groups provide peer support and shared experiences, which can enhance emotional well-being and provide a network of encouragement for patients dealing with chronic pain.^552^ Current management of DPN-related complications primarily relies on foot examinations, fall prevention, and psychological support; however, there are shortcomings in implementing patient education, multidisciplinary collaboration, and psychological interventions. Future studies should optimize these management strategies and strengthen multimodal interventions to reduce complication rates and improve patient quality of life.

### Treatment of DAN

DAN is a serious complication of diabetes mellitus that affects the ANS, leading to dysfunction in multiple organ systems. Currently, there is no effective etiological treatment for DAN, and management primarily focuses on alleviating clinical symptoms and improving patients’ quality of life. Specific abnormal clinical presentations require targeted interventions to mitigate their impact on daily functioning.

#### CAN

Management strategies for severe CAN, particularly orthostatic hypotension, focus on alleviating symptoms, extending standing time, enhancing physical capacity, and improving overall activity levels rather than solely increasing standing blood pressure. Non-pharmacological interventions include medication adjustment.^553^ Fluid and salt intake should be encouraged to increase plasma volume, with a high-sodium diet potentially raising standing blood pressure and alleviating symptoms.^554^ Physical countermeasures encompass the use of compression garments to reduce venous pooling in the lower extremities and abdomen, as well as teaching physical maneuvers to increase venous return when standing.^555,556^

Lifestyle modifications include head-up tilt sleeping, which elevates the head of the bed by 10–30° to reduce nocturnal diuresis and improve morning orthostatic tolerance, and advising patients to avoid triggers such as prolonged standing, sudden postural changes, high ambient temperatures, and large meals that may exacerbate symptoms.^557,558^ Dietary adjustments for postprandial hypotension recommend small, frequent meals with low carbohydrate content to minimize splanchnic blood pooling after eating.^559^ When non-pharmacological measures are insufficient, pharmacological treatments may be considered. These include midodrine, an alpha-1 adrenergic agonist approved by the FDA for symptomatic orthostatic hypotension that induces vasoconstriction to increase peripheral resistance and standing blood pressure;^560^ droxidopa, a norepinephrine precursor that enhances sympathetic tone by increasing norepinephrine levels and improving orthostatic symptoms;^561^ fludrocortisone, a mineralocorticoid that promotes sodium and water retention to expand plasma volume, though it must be used cautiously due to the risk of supine hypertension and hypokalemia;^562^ pyridostigmine, an acetylcholinesterase inhibitor that augments sympathetic ganglion transmission to increase vascular resistance during standing without significantly affecting supine blood pressure.^563^ Monitoring and considerations include dose titration, initiating medications at low doses and gradually increasing to the maximum tolerated dose while monitoring efficacy and adverse effects;^564^ careful monitoring for supine hypertension, which occurs in approximately 50% of patients with orthostatic hypotension to balance treatment benefits with potential risks;^565^ combination therapy, where if monotherapy is ineffective, medications with different mechanisms of action may be combined.^566^

#### Gastrointestinal autonomic neuropathy

Gastrointestinal autonomic neuropathy can affect any part of the gastrointestinal tract, leading to symptoms such as gastroparesis, constipation, and diarrhea. Management focuses on symptom relief and improving gastrointestinal motility. Dietary modifications are essential, including encouraging patients to consume small, frequent meals to reduce gastric distension and improve emptying, adopting a low-fiber, low-fat diet to minimize delays in gastric emptying, and incorporating liquid nutrients that are easier to digest.^567^ A comprehensive medication review is necessary to identify and discontinue medications that may impair gastric motility.^568^ Pharmacological treatments for gastroparesis include metoclopramide, a prokinetic and antiemetic agent that stimulates gastric emptying by antagonizing dopamine receptors; however, its use is limited by the risk of serious side effects like tardive dyskinesia, necessitating the lowest effective dose for the shortest duration.^569^ Erythromycin enhances gastric contractions and may be beneficial in the short term, although its long-term use is limited by tachyphylaxis and potential antibiotic resistance.^570^ Domperidone is not FDA-approved but is available in some countries under investigational protocols.^571^ Other interventions include gastric electrical stimulation^572^ and optimizing glycemic control.^573^

#### Genitourinary autonomic neuropathy

Genitourinary autonomic neuropathy in diabetes patients can lead to significant dysfunctions, such as ED, neurogenic bladder, and female sexual dysfunction, each requiring targeted management strategies to improve patient outcomes. ED is commonly managed with first-line treatments, including phosphodiesterase type 5 (PDE5) inhibitors (sildenafil, tadalafil, and vardenafil), which enhance nitric oxide-mediated vasodilation to facilitate erections and are effective in many diabetes patients.^574^ Second-line treatments involve intracavernosal injections of alprostadil for those unresponsive to oral medications and the use of vacuum erection devices, which are non-invasive methods to engorge the penis with blood.^575,576^ Third-line treatments include penile prosthesis implantation for patients who fail or are intolerant to other therapies.^577^ Conversely, non-pharmacological interventions, such as bladder training with scheduled voiding and pelvic floor exercises, along with intermittent catheterization, are essential to prevent urinary retention and reduce infection risks.^578^ Pharmacological treatments include cholinergic agonists, such as bethanechol, to stimulate detrusor muscle contraction, antimuscarinic agents, such as oxybutynin or tolterodine, for overactive bladder symptoms, and beta-3 agonists, such as mirabegron, to relax bladder smooth muscle during the storage phase.^579,580^ Advanced therapies encompass botulinum toxin injections to reduce bladder overactivity and sacral neuromodulation.^581,582^ Female sexual dysfunction associated with CAN is managed by optimizing glycemic control to enhance nerve function, promptly treating urinary and genital infections, using lubricants and moisturizers to alleviate vaginal dryness, and implementing pelvic floor therapy to strengthen pelvic muscles.^583–586^ Additionally, psychological support through counseling and sex therapy addresses psychological factors, such as depression, anxiety, and relationship issues, thereby improving sexual function and overall quality of life.^550,551^

#### Sudomotor dysfunction

Gustatory sweating, a form of sudomotor dysfunction, is characterized by excessive sweating of the face and neck during eating, especially in response to spicy or acidic foods. Effective management strategies for gustatory sweating include topical anticholinergic agents and botulinum toxin injections. Glycopyrrolate cream at a concentration of 0.5% is topically applied to reduce sweating by blocking acetylcholine receptors in sweat glands, thereby decreasing perspiration.^587^ When topical treatments are insufficient, Botulinum Toxin Type A injections into the affected areas inhibit acetylcholine release at neuromuscular junctions, resulting in a significant reduction in sweating for several months.^588^

Current treatments for DAN focus primarily on alleviating symptoms and improving quality of life but lack effective etiological therapies; some treatments have limited efficacy. Future studies should develop more targeted therapies and optimize comprehensive management strategies to enhance patient outcomes and quality of life.

## DN and associated complications

Diabetic foot disease is significantly exacerbated in patients with DPN due to reduced sensation and impaired wound healing, elevating the risk of foot ulcers and infections and potentially leading to lower-limb amputations. A recent systematic review and meta-analysis highlighted the high global prevalence of diabetic foot ulceration among DPN patients, emphasizing the critical need for early detection and comprehensive management strategies to prevent severe outcomes.^589^ DN and DPN share a reciprocal relationship, both arising from prolonged hyperglycemia and common pathophysiological mechanisms, such as oxidative stress, inflammation, and endothelial dysfunction. Patients with DPN are at high risk of developing chronic kidney disease; renal impairment can further exacerbate neuropathic symptoms through the accumulation of uremic toxins that adversely affect nerve function.^590^ Diabetic retinopathy is also closely associated with DPN, with shared risk factors, such as poor glycemic control, hypertension, and dyslipidemia contributing to the development of both microvascular complications. A cross-sectional study found a significant correlation between the severity of DPN and the progression of diabetic retinopathy, suggesting overlapping pathogenic pathways and the need for integrated management approaches.^42^ Additionally, DPN is linked to an increased risk of cardiovascular disease (CVD). Autonomic neuropathy, a component of DPN, can lead to abnormalities in HRV and elevate the risk of silent myocardial ischemia, particularly in patients with T2DM. CAN is associated with a higher incidence of cardiovascular events and mortality among diabetes patients.^5^

The coexistence of DPN with other diabetes-related complications necessitates a comprehensive approach to patient care, especially for elderly patients. Holistic management involves routine screening for DPN and associated complications.^543^ Multidisciplinary care models involving endocrinologists, neurologists, podiatrists, nephrologists, ophthalmologists, cardiologists, dietitians, and physical therapists improve patient outcomes by addressing all aspects of health comprehensively, thereby reducing hospitalizations and healthcare costs.^591^ In elderly patients, managing DPN alongside other chronic conditions becomes more complex due to polypharmacy, potential drug interactions, and physical and cognitive limitations. This emphasizes the importance of individualized care plans for elderly diabetes patients, balancing glycemic targets with the risk of hypoglycemia and other adverse effects.^592^ Furthermore, patient education and support are crucial for effective diabetes management. Indeed, a systematic review highlighted that psychological support and patient education are integral components of effective diabetes management.^593^ Current research on DN and its associated complications remains limited in terms of comprehensive management and multidisciplinary collaboration, and the interactions between different complications remain poorly understood. It is necessary to enhance the development of integrated treatment strategies, promote interdisciplinary cooperation, and fully investigate the shared pathophysiological mechanisms to improve patient outcomes and quality of life.

## Emerging therapies and future drug prospects

DN is a complex and multifactorial complication of diabetes mellitus, often progressing despite optimal glycemic control. Current treatments primarily focus on symptom management, with limited efficacy in halting or reversing disease progression. This underscores the urgent need for novel therapies that target the underlying mechanisms of DN.

### Unmet clinical needs

Despite stringent glycemic control, several patients with T2DM continue to experience the progression of DN. Existing cardiovascular treatments, such as aspirin and antioxidants, primarily address comorbid conditions but show limited efficacy in treating DN.^5^ Additionally, patients often develop tolerance to analgesics, necessitating prolonged high-dose therapies and combination regimens.^17^ The COMBO-DN study found that drug combinations did not significantly outperform high-dose monotherapy.^594^ The OPTION-DM study determined which monotherapy and combination therapy of amitriptyline, duloxetine, and pregabalin are the most clinically efficacious; the authors showed that all three treatment pathways and monotherapies had similar analgesic efficacy.^595^

Therefore, treatments that restore normal neurological function, or at least slow disease progression by targeting specific pathogenic mechanisms, remain warranted. Restoring normal neurological function, or at least slowing disease progression, should be the focus of new therapies to address DN. Furthermore, defining the optimal treatment duration and enhancing analgesic strategies remain necessary. New pain management medications should exhibit higher response rates than existing options and aim to target specific pain pathways while improving understanding of dosing regimens and treatment cycles.^5^

### Novel targeted drugs

**Fig. 6:**
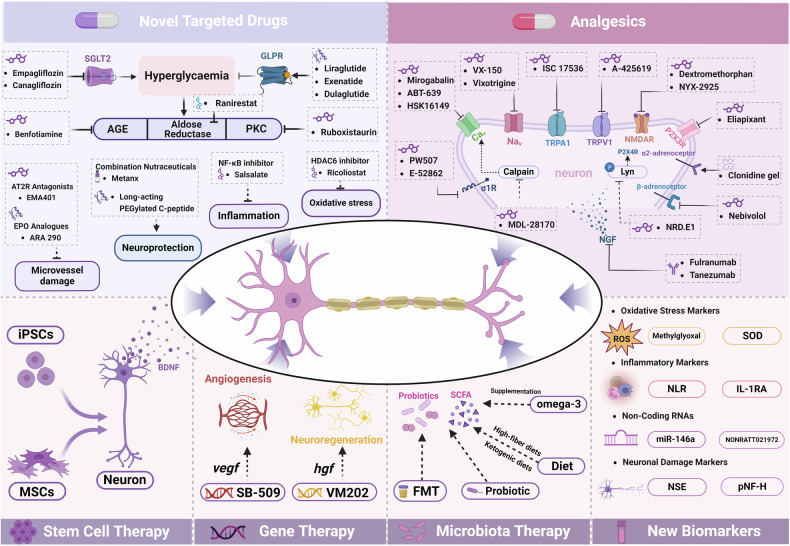
Emerging therapeutic strategies for diabetic neuropathy. A range of repurposed drugs and preclinically tested lead compounds are under investigation for the management of DN. Newer targeted therapies, including SGLT2 inhibitors and GLP receptor agonists, are being integrated into the prevention and treatment of DN. Additionally, novel ion channel modulators, such as voltage-gated sodium and calcium channel blockers, TRPA1 and TRPV1 antagonists, NMDAR antagonists, and P2X3 receptor antagonists, are employed to mitigate pain in patients with PDPN. Innovative approaches such as stem cell therapy, gene therapy, and fecal microbiota transplantation are also being explored to enhance DN management. Concurrently, emerging molecular biomarkers, including non-coding RNAs and neuronal injury markers, are under investigation to improve the clinical diagnosis and prognosis of DN. AGEs advanced glycation end products, AT2R angiotensin II type 2 receptor, BDNF brain-derived neurotrophic factor, DN diabetic neuropathy, FMT fecal microbiota transplantation, EPO erythropoietin, GLPR glucose-dependent insulinotropic polypeptide receptor, HDAC6 histone deacetylase 6, HGF hepatocyte growth factor, IL-1RA interleukin-1 receptor antagonist, iPSC induced pluripotent stem cells, MSCs mesenchymal stem cells, NF-κb nuclear factor kappa-light-chain-enhancer of activated B cells, NGF nerve growth factor, NLR neutrophil-to-lymphocyte ratio, NMDAR N-methyl-D-aspartate receptor, NSE neuron-specific enolase, PDPN painful diabetic peripheral neuropathy, pNF-H phosphorylated neurofilament heavy chain, P2X3R P2X receptor subtype 3, P2X4R P2X receptor subtype 4, SCFA short-chain fatty acids, SGLT2 sodium-glucose transport protein 2, SOD superoxide dismutase, TRPV1 transient receptor potential vanilloid 1, TRPA1 transient receptor potential ankyrin 1, TRPM8 transient receptor potential melastatin 8, VEGF vascular endothelial growth factor

Advancements in understanding the pathophysiological mechanisms of DN have led to the development of novel targeted therapies aimed at specific molecular pathways involved in nerve damage and pain transmission (Table 1 and Fig. 6).

**Table 1:** Emerging drugs in diabetic neuropathy

Agent	Mechanism of action/target	Clinical trials’ number	Participants (n)	Phase	Main outcome
Empagliflozin^598^	SGLT2 inhibitor	NCT05977465	50	2	Empagliflozin showed a significant improvement in the electrophysiological studies and a significant decrease in the pain score and the mean serum levels of NSE and MDA.
Canagliflozin^601^	SGLT2 inhibitor	NCT02554126	4401	2	Canagliflozin did not affect the risk of neuropathy events.
Liraglutide^602^	GLPR agonist	NCT02138045	39	2	Neuronal function was unaltered at the central, autonomic or peripheral level.
Exenatide^604^	GLPR agonist	NCT00855439	46	2	There were no statistically significant treatment group differences in the prevalence of CCN, IENFD and nerve conductions.
Dulaglutide^605^	GLPR agonist	UMIN000044264	120	2	Diabetes treatment-related quality of life scores associated with pain and gastrointestinal symptoms were also superior in the dulaglutide group.
Ranirestat^606^	Aldose reductase inhibitor	NCT00101426	549	3	Ranirestat influenced motor nerve function in mild to moderate DSPN.
Benfotiamine^608^	Anti-AGEs	DRKS00014832	57	2	Undergoing.
Ruboxistaurin^609^	PKC inhibitor	NCT00190970	52	2	Ruboxistaurin enhanced skin microvascular blood flow at the distal calf, reduced sensory symptoms, improved the quality of life, and was well tolerated.
Actovegin^611^	PARP inhibitor	NCT00483730	567	3	Actovegin improved neuropathic symptoms, VPT, sensory function, and quality of life.
VX-150	Na_V_1.8 inhibitor	NCT03304522	34	2	VX-150 could reduce pain score caused by small fiber neuropathy.
Vixotrigine(BIIB074)^614^	Voltage-Gated sodium channel blocker	NCT03339336	265	2	Vixotrigine showed significant improvement in diabetes patients with painful small fiber neuropathy.
Mirogabalin^616^	Calcium channel modulator	NCT02318706	834	3	Mirogabalin showed statistically significant pain relief (vs. placebo) in Asian PDPN patients.
ABT-639^617^	Calcium channel blocker	NCT01345045	194	2	There was no difference (*p* = 0.582) in pain alleviation between ABT-639 and placebo and ABT-639 showed no safety signals.
HSK16149^618^	Calcium channel blocker	NCT04647773	725	3	Efficacy of HSK16149 capsules was superior to placebo in all groups for relieving PDPN and appeared well tolerated.
Eliapixant (BAY 1817080)^620^	Selective P2X3 antagonist	NCT04641273	135	2	Eliapixant in patients with diabetic neuropathic pain (DNP) did not translate to any relevant improvement in different pain intensity outcomes compared with placebo.
MDL-28170^621^	Calpain inhibitor				MDL-28170 reduced mechanical allodynia and thermal hyperalgesia and improved nerve conduction velocity.
ISC 17536^622^	TRPA1 antagonist	NCT01726413	138	2	ISC 17536 had a statistically and clinically significant improvement effects on pain.
Dextromethorphan^623^	NMDAR antagonist	NCT00695565	45	1	Dextromethorphan was effective in a dose-dependent manner in selected patients with painful diabetic neuropathy.
NYX-2925^624^	NMDAR antagonist	NCT04146896	228	2	NYX-2925 did not achieve a statistically significant difference compared with placebo in the primary endpoint, but improved pain scores in patients with DPN in a phase 2 study.
NRD.E1^626^	Regulation of the phosphorylation of Lyn tyrosine kinase	NCT02345291	88	2	NRD.E1 induced a clinically relevant pain reduction and it was well tolerated.
EMA401^627^	AT2R antagonist	NCT03094195 NCT03297294	45 49	2	EMA401 showed analgesic efficacy and safety in two studies.
ARA 290^628^	Erythropoietin analog	NTR3858	50	2	ARA 290 benefited metabolic control and neuropathic symptoms in patients with T2DM.
SB-509^653^	PhVEGF165 gene transfer	IND577	29	2	This treatment ameliorated neuropathic symptoms.
Buprenorphine^629^	Partial μ-opioid receptor agonist	ACTRN12609000647235	186	2	Transdermal buprenorphine, when tolerated, was an effective therapy for PDPN.
PL37^631^	Dual enkephalinase inhibitor	EudraCT Number:2013-004876-37	120	2	Early termination.
Nebivolol^633^	Beta-blocker	NCT06201611	120	3	Undergoing.
Clonidine gel^634^	α2-adrenoceptor agonist	NCT00695565	180	2	Topical clonidine gel significantly reduced the level of foot pain in painful diabetic neuropathy.
LX-9211^636^	AAK1 inhibitor	NCT04455633	319	2	LX-9211 improved PDPN.
PW507^637^	σ1R antagonist				PW507 demonstrated significant efficacy in alleviating mechanical allodynia and thermal hyperalgesia following both acute and chronic (2-week) administration.
E-52862^638^	σ1R antagonist				E-52862 reversed neuropathic and vascular signs in the ZDF rat.
Fulranumab^639^	Human monoclonal antibody against NGF	NCT00993018	77	2	Fulranumab treatment resulted in dose-dependent efficacy and was generally well tolerated.
Tanezumab^640^	Humanized monoclonal antibody against NGF	NCT01087203 NCT00568321	73 96	2	Tanezumab provided effective pain reduction in DPN and no new safety concerns were observed.
Salsalate^641^	NF-κB inhibitor	NCT01480297	8	2	Salsalate could improve nerve conduction velocity.
ALC^643^	Neuroprotective and neurotrophic effects	NCT05319275	907	3	ALC significantly improved symptoms and somatosensory symptoms.
Long-acting PEGylated C-peptide^645^	Improving nerve blood flow and nerve conduction velocity	NCT01681290	250	2	A significant reduction in VPT in patients treated with C-peptide compared with placebo.
Ricoliostat^647^	HDAC6 inhibitor	NCT03176472	282	2	Changes in average daily pain were not different between the ricolinostat and placebo groups.
Metanx^343^	Oxidative nitrosative stress	NCT00726713	214	2	Results showed improvements in neuropathic symptoms and quality of life.
VM202^651^	plasmid DNA encoding HGF isoform	NCT02427464	500	3	VM202 demonstrated significant pain reduction and was well tolerated.
Seal oil omega-3 polyunsaturated fatty acids^656^	Nerve structure and function protection	NCT02034266	40	2	ω-3 supplementation was associated with increase in corneal nerve fiber length in T1DM.

*AGEs* advanced glycation end products, *ALC* acetyllevocarnitine hydrochloride, *AT2R* angiotensin II type 2 receptor, *CCN* confirmed clinical neuropathy, *DNP* diabetic neuropathic pain, *DSPN* distal symmetric polyneuropathy, *GLPR* glucose-dependent insulinotropic polypeptide receptor, *HDAC6* histone deacetylase 6, *HGF* hepatocyte growth factor, *IENFD* intraepidermal nerve fiber density, *MDA* malondialdehyde, *NF-κb* nuclear factor kappa-light-chain-enhancer of activated B cells, *NGF* nerve growth factor, *NMDAR* N-methyl-D-aspartate receptor, *NSE* neuron-specific enolase, *PDPN* painful diabetic peripheral neuropathy, *P2X3R* P2X receptor subtype 3, *σ1R* sigma 1 receptor, *SGLT2* sodium-glucose transport protein 2, *TRPA1* transient receptor potential ankyrin 1, *T1DM* types 1 diabetes mellitu, *T2DM* types 2 diabetes mellitu, *VEGF* vascular endothelial growth factor, *VPT* vibration perception threshold, *ZDF* zucker diabetic fatty

**SGLT2 inhibitors**

- **Empagliflozin:** Empagliflozin is a novel SGLT2 inhibitor, the underlying mechanisms of which include ameliorating hyperglycemia, preventing the development of endothelial dysfunction, reducing oxidative stress, and improving metabolic profiles.^596^ Empagliflozin can prevent the loss of skin intraepidermal nerve fibers and mesangial matrix expansion^597^ and a potential therapeutic effect on DPN. In a phase 2 study, comparing the results of the empagliflozin arm to the control arm showed a significant improvement in the electrophysiological studies and a significant decrease in the pain score and the mean serum levels of neuron-specific enolase and malondialdehyde in patients with DPN.^598^
- **Canagliflozin:** Aa an SGLT2 inhibitor for the treatment of T2DM,^599^ canagliflozin reduces obesity-related inflammation in neural tissue and skeletal muscle.^600^ In a multicenter, randomized, double-blind, placebo-controlled trial, canagliflozin did not affect the risk of neuropathy events.^601^ Future large, randomized studies with prespecified neurologic endpoints are warranted to determine the effects of SGLT2 inhibitors on DN.

**GLP-1 receptor agonists**

- **Liraglutide:** Liraglutide has shown anti-inflammatory properties. In a randomized, double-blinded, placebo-controlled trial, compared with placebo, liraglutide reduced IL-6 with concomitant numerical reductions in other proinflammatory cytokines; however, neuronal function was unaltered at the central, autonomic, or peripheral level.^602^
- **Exenatide:** Exenatide may have direct neuroprotective and neurotrophic effects independent of its glycemic effects.^603^ In an 18-month proof-of-concept open-label randomized study, no statistically significant treatment group differences in the prevalence of confirmed clinical neuropathy (CCN), IENFD, and nerve conductions were observed.^604^
- **Dulaglutide:** In a randomized, parallel-group, multicenter, open-label trial, diabetes treatment-related quality of life scores associated with pain and gastrointestinal symptoms were superior in the dulaglutide group.^605^

**Aldose reductase inhibitor**

- **Ranirestat**: Ranirestat is a potent aldose reductase inhibitor that reduces nerve sorbitol accumulation. A clinical trial has shown that ranirestat affects motor nerve function in mild to moderate DPN.^606^ It failed to show a statistically significant difference in sensory nerve function relative to placebo.

**Anti-AGEs**

- **Benfotiamine**: Benfotiamine, a lipid-soluble derivative of thiamine. The ability of benfotiamine to inhibit three major pathways (the hexosamine pathway, the AGE formation pathway, and the diacylglycerol (DAG)-PKC pathway) simultaneously might be clinically useful in preventing the development and progression of diabetic complications.^233^ In a double-blind, placebo-controlled, phase-3 study, benfotiamine can reduce pain and is well tolerated.^607^ A randomized, double-blind, placebo-controlled trial over 12 months to assess the effects of benfotiamine on morphometric, neurophysiological, and clinical measures in patients with T2DM with symptomatic polyneuropathy is undergoing.^608^

**PKC inhibitor**

- **Ruboxistaurin:** Ruboxistaurin targets the PKC-β isoform implicated in diabetic microvascular complications. In a 6-month, randomized, double-masked, placebo-controlled study, ruboxistaurin enhanced skin microvascular blood flow at the distal calf, reduced sensory symptoms, improved the quality of life, and was well tolerated in DPN patients.^609^

**PARP inhibitor**

- **Actovegin:** Actovegin ameliorated sensory nerve conduction velocity (SNCV) and IENFD of experimental DN via mechanisms involving suppression of PARP activation.^610^ Actovegin improved neuropathic symptoms, vibration perception threshold (VPT), sensory function, and quality of life in patients with DPN in a multicenter, randomized, double-blind trial.^611^

**Voltage-gated sodium and calcium channel blockers**

- **VX-150:** A pro-drug of a highly selective NaV1.8 inhibitor. VX-150 induces analgesia without compromising subject safety in a phase 1 study. VX-150 also has a significant effect on cold pressure pain thresholds and heat pain thresholds.^612^ A phase 2 study showed that VX-150 could reduce pain scores caused by SFN.
- **Vixotrigine (BIIB074)**: Vixotrigine is a state-dependent sodium channel blocker that targets hyperexcitable neurons involved in pain transmission. A phase 2a trial in patients with trigeminal neuralgia, vixotrigine may provide a potential treatment option, although it did not show a statistically significant advantage.^613^ In a phase 2 study, vixotrigine was investigated for the treatment of idiopathic or diabetes-associated painful SFN. Vixotrigine showed significant improvement in diabetes patients with painful SFN.^614^ Its efficacy in painful DN is currently under investigation.
- **Mirogabalin**: Mirogabalin selectively binds to the α2δ-1 subunit of VGCCs, inhibiting overactive neurons. Mirogabalin provides effective pain relief with improved tolerability compared to traditional agents like pregabalin.^615^ In a phase-3 study, mirogabalin showed statistically significant pain relief (vs. placebo) in Asian PDPN patients.^616^
- **ABT-639**: A peripherally acting highly selective T-type Cav3.2 calcium channel blocker. A phase 2 clinical trial reported no difference in pain alleviation in patients with DN pain between ABT-639 and placebo and that ABT-639 showed no safety signals.^617^
- **HSK16149:** HSK16149 is a novel ligand α2-δ subunit of VGCC. A Phase-3 randomized clinical trial showed that the efficacy of HSK16149 capsules was superior to placebo in all groups for relieving PDPN and appeared well tolerated.^618^ Forty- and 80-mg/d doses of HSK16149 were recommended for treating patients with PDPN in China. On May 20, 2024, HSK16149 was approved by the National Food and Drug Administration (NMPA) of China for treating PDNP in adults.

**Selective P2X3 antagonist**

- **Eliapixant (BAY 1817080):** ATP-dependent P2X3 receptors play a fundamental role in nerve fiber sensitization and pathological pain pathways.^619^ Eliapixant in patients with DNP did not translate to any relevant improvement in different pain intensity outcomes compared with placebo in phase 2a of the PUCCINI study.^620^

**Calpain inhibitor**

- **MDL-28170:** Calpain plays an important role in the pathophysiology of neurological complications. In DRG neurons of STZ-induced diabetes rats, intraperitoneal administration of MDL-28170 has shown beneficial effects in alleviating DN via modulation of TTX-R Na (+) channel kinetics and reducing oxidative stress and neuroinflammation in STZ-induced diabetes rats.^621^

**TRPA1 antagonist**

- **ISC 17536:** ISC 17536 is a potent and selective antagonist targeting TRPA1. In a randomized, double-blind, placebo-controlled trial, patients with preserved small nerve fiber function as defined by QST had a statistically and clinically significant improvement in pain.^622^

**NMDA receptor antagonists**

- **Dextromethorphan:** NMDA receptor antagonists may mitigate central sensitization and enhance descending inhibitory pathways involved in neuropathic pain. Dextromethorphan and memantine are being explored for their potential benefits in DPN. In efficacy and dose-response trials, no comparison with placebo reached statistical significance in the efficacy trial.^623^ Dextromethorphan is effective in a dose-related fashion in selected patients with painful DN.
- **NYX-2925:** A novel co-agonist to glutamate at the NMDA receptor. A first-in-human, randomized, double-blind study of safety and pharmacokinetics in adults suggested that NYX-2925 was safe and well tolerated.^624^ NYX-2925 did not achieve a statistically significant difference with placebo in the primary endpoint; however, it improved pain scores in patients with DPN in a phase 2 study.

**Regulation of the phosphorylation of Lyn tyrosine kinase**

- **NRD.E1:** Presumably, the mechanism of action of NRD.E1 involves regulation of the phosphorylation of Lyn tyrosine kinase, a key step in P2X4 receptor upregulation in neuropathic pain induced by nerve injury.^625^ NRD.E1 is a novel non-opioid therapeutic that is being developed for the treatment of PDPN.^626^ In phase 2a, randomized, dose-finding, proof-of-concept study, NRD.E1 induced a clinically relevant pain reduction and was well tolerated.

**Angiotensin II type 2 receptor antagonists**

- **EMA401:** EMA401 has been investigated for its potential to reduce inflammation in peripheral tissues and decrease peripheral sensitization. EMA401 showed analgesic efficacy and safety in two randomized, double-blind, phase 2 studies of postherpetic neuralgia and painful DN. Although both studies were terminated early due to long-term preclinical hepatotoxicity.^627^

**Erythropoietin analogs**

- **ARA 290** is an 11-amino acid peptide derived from erythropoietin that interacts with the innate repair receptor to promote anti-inflammatory and neuroprotective effects. A double-blind, placebo-controlled, investigator-initiated clinical trial showed that ARA 290 benefited both metabolic control and neuropathic symptoms in patients with T2DM.^628^

**μ-opioid receptor agonis**

- **Buprenorphine:** Transdermal buprenorphine was evaluated in a randomized, double-blind, parallel-group, placebo-controlled trial. Pain reduction by 30% was more frequent in the buprenorphine vs. placebo group, but many patients withdrew from the study.^629^ Transdermal buprenorphine, when tolerated, is an effective therapy for PDPN.

**Dual enkephalinase inhibitor**

- **PL37:** An orally administered dual inhibitor of enkephalinases.^630^ PL37 can increase the concentration of enkephalins peripherally and enhance the endogenous opiodergic system. PL37 showed a significant analgesic effect in animal models that can reduce neuropathic pain without the possible side effects of opiates.^631^ However, similar results were not found in a 4-week phase 2a study involving patients with PDPN. This trial was prematurely discontinued, and the results have not been published.

**β-adrenoceptor antagonist**

- **Nebivolol:** Nebivolol is a β-adrenoceptor antagonist with nitric oxide enhancement, vasodilation, and antioxidant effects. Nebivolol can alleviate thermal hyperalgesia in diabetes rats by releasing NO activity.^632^ Nebivolol prevents disease progression in patients with DPN, probably through its dual effects as an antioxidant and NO enhancer.^633^ A 3-arm, open-label, stratified randomized controlled trial with a blinded endpoint assessment to evaluate a NO generator (Nebivolol) as a disease-modifying medication in DPN is undergoing.

**α2-adrenoceptor agonists**

- **Clonidine gel:** A randomized, double-blind, placebo-controlled, parallel-group, multicenter trial showed that topical clonidine gel significantly reduced the level of pain in subjects with DN in whom there are functional (and possibly sensitized) nociceptors in the affected skin.^634^

**AAK1 inhibitor**

- **LX-9211:** Junctional protein 2-associated kinase 1 (AAK1) is widely expressed in the brain and spinal cord and is a viable target for the treatment of neuropathic pain, and BMS-986176/LX-9211 is a novel class of bis(iso)aryl ethers that are highly selective, CNS-permeable, and potent AAK1 inhibitors.^635^ A double-blind, randomized, placebo-controlled trial of LX-9211 for pain relief and safety in DPN initially found that LX-9211 improved PDPN.^636^

**Sigma 1 receptor (σ1R) antagonists**

- **PW507:** σ1R plays a key role in regulating cellular processes associated with pain modulation. In preclinical rat models of STZ-induced DN, PW507 demonstrated significant efficacy in alleviating mechanical allodynia and thermal hyperalgesia following acute and chronic (2-week) administration without inducing tolerance and visual evidence of toxicity.^637^ This report offers evidence for the potential use of PW507 as a promising therapeutic option for PDN.
- **E-52862:** E-52862 administration reverses neuropathic (behavioral and electrophysiological) and vascular signs in the Zucker diabetic fatty (ZDF) rat.^638^ Blocking σ1R prevents DN in rats and is a potentially effective treatment for peripheral neuropathies in diabetes patients.

**Monoclonal antibody against NGF**

- **Fulranumab:** Fulranumab is a human monoclonal antibody against NGF. A phase 2, double-blind, placebo-controlled trial randomized patients with moderate-severe PDPN to subcutaneous fulranumab or placebo.^639^ Despite early study termination (clinical hold) by the US Food and Drug Administration, fulranumab treatment resulted in dose-dependent efficacy and was generally well tolerated.
- **Tanezumab:** Tanezumab is a humanized monoclonal antibody against NGF. Two RCTs examined the efficacy of tanezumab vs. placebo in patients with DPN or postherpetic neuralgia.^640^ In the DPN study, tanezumab provided effective pain reduction in DPN, with no safety concerns observed.

**Anti-inflammatory agents**

- **Salsalate:** NF-κB plays a crucial role in the transcription of proinflammatory cytokines contributing to nerve damage. Salsalate, an NF-κB inhibitor, can improve fasting plasma glucose, reduce HbA1c levels, and reduce proinflammatory markers in T2DM patients.^641^ A phase 2 study showed that salsalate can improve NCV.

**Acetyllevocarnitine hydrochloride (ALC)**

- **ALC**: ALC has neuroprotective and neurotrophic effects on the PNS.^642^ In a multicenter, randomized, double-blind, placebo-controlled phase-3 clinical trial conducted in China, acetyllevocarnitine hydrochloride treatment for 24 weeks significantly improved symptoms and somatosensory symptoms in patients with DPN, with a treatment effect of 65% higher than placebo.^643^

**Long-acting PEGylated C-peptide**

- **C-peptide** has neuroprotective properties in T1DM, improving nerve blood flow and NCV and increasing expression of beneficial neurotrophic factors.^644^ A long-acting form of C-peptide has been developed to enhance its stability and half-life. A 12-month clinical trial demonstrated a significant reduction in VPT in patients treated with C-peptide compared with placebo.^645^ However, the primary endpoint of improved SNCV was not met, and further studies remain uncertain.

**HDAC6 inhibitor**

- **Ricoliostat:** HDAC6 inhibitors can promote the degradation of protein aggregates or protect neurons from oxidative stress, hence can be used as possible drugs for treating peripheral neuropathy.^646^ Ricoliostat, a selective HDAC6 inhibitor, failed to ameliorate neuropathic pain in patients with DPN when administered 120 mg/kg daily of ricolinostat for 12 weeks compared with placebo.^647^ However, ricoliostat has a favorable safety and tolerability profile and does not exclude the possibility that longer treatment may have a therapeutic effect on DPN or other small fiber neuropathies.

**Combination nutraceuticals**

- **Metanx**: Metanx is a prescription medical food containing L-methylfolate, methylcobalamin, and pyridoxal-5′-phosphate. Metanx ingredients counteract endothelial NO synthase uncoupling and oxidative stress in vascular endothelium and peripheral nerve.^344^ A randomized trial showed improvements in neuropathic symptoms and quality of life measures in patients with DPN. However, further studies are needed to confirm its efficacy and long-term benefits.^343^

### Stem cell therapy

Stem cell therapy offers a regenerative approach to treating DPN by promoting nerve repair and regeneration.

- **Mesenchymal stem cells (MSCs)**: MSCs can differentiate into neuronal cells and secrete neurotrophic factors like NGF and BDNF.^648^ Preclinical studies have shown that MSC transplantation improves nerve function and reduces neuropathic pain in diabetic animal models^649^.
- **Induced pluripotent stem cells (iPSCs)**: iPSCs derived from patients’ cells offer a personalized therapy option. Research indicates that chemogenetic stimulation of hiPSC-derived neural cells enhances cell activity and neuron-to-neuron interactions in vitro.^650^ Clinical trials are ongoing to evaluate the safety and efficacy of stem cell therapies in patients with DPN.

### Gene therapy

Gene therapy targets the underlying genetic and molecular mechanisms contributing to DPN.

- **VM202**: VM202 is a plasmid DNA encoding human hepatocyte growth factor (HGF) isoform. HGF promotes angiogenesis and neurogenesis, potentially improving nerve regeneration. A phase-3 study was conducted to evaluate the safety and efficacy of VM202 in DPN. VM202 demonstrated significant pain reduction and was well tolerated, although not all endpoints were met.^651,652^
- **SB-509 (PhVEGF165 gene transfer)**: VEGF gene therapy aims to enhance angiogenesis and nerve repair. An open-label trial used VEGF165 gene transfer as an intra-muscular injection in the ischemic thigh in patients with critical ischemia with/without DM; the treatment ameliorated neuropathic symptoms.^653^

### Microbiota therapy

Emerging evidence suggests that the gut microbiota plays a role in the pathogenesis of DPN.

- **FMT**: FMT involves transferring gut microbiota from healthy donors to patients to restore microbial balance. A randomized, double-blind, and placebo-controlled trial demonstrated that FMT reduced inflammatory markers and improved neuropathic symptoms in patients with DPN.^654^
- **Probiotic supplementation**: A randomized controlled trial has shown that probiotic supplementation among patients with diabetes significantly decreased biomarkers of inflammation and oxidative stress.^655^
- **Omega-3 supplementation:** In a single-arm, open-label trial of seal oil ω-3 PUFA supplementation (composed of eicosapentaenoic acid, docosapentaenoic acid, and docosahexaenoic acid) for 1 year in patients with T1DM and diabetic sensorimotor polyneuropathy, a 29% improvement in the primary endpoint of corneal nerve fiber length was observed. However, there was no change in nerve conduction or sensory function.^656^
- **Dietary interventions**: High-fiber and ketogenic diets have been used to treat chronic pain conditions. High-fiber diets, possibly through gut microbiota modulation, increase short-chain fatty acid production,^657^ while a ketogenic diet reduces metabolic syndrome-induced allodynia and increases epidermal axon density in mice.^658^

### Advanced drug delivery systems

Innovations in drug delivery aim to enhance the efficacy and specificity of therapeutic agents for DN. Nanometer-sized drug carriers can improve bioavailability, achieve controlled release, enhance the selectivity of therapeutic agents, minimize side effects, and optimize treatment outcomes.^659^ Nanotechnology-based strategies, such as lipid and polymeric nanoparticles, have been widely investigated for their potential application in improving drug delivery to enhance action to the target area to mitigate diabetic neuropathic pain.^660^ Nanotechnology-based interventions offer promising prospects in diabetes management. Advanced formulations allow for sustained release of therapeutic agents, reducing dosing frequency and improving patient compliance.^661^

### Biomarkers and diagnostic advances

Identifying reliable biomarkers for DN is crucial for early diagnosis and monitoring of disease progression.

- **Oxidative stress markers:** Elevated levels of methylglyoxal (MG) and decreased superoxide dismutase (SOD) activity are associated with DPN, reflecting increased oxidative stress.^225^
- **Inflammatory markers:** Neutrophil-to-lymphocyte ratio (NLR) and IL-1 receptor antagonist (IL-1RA) levels correlate with DPN severity, indicating the role of inflammation.^662^
- **Non-Coding RNAs:** Altered expression of miR-146a has been implicated in DPN pathogenesis by modulating inflammatory pathways.^663^ Long non-coding RNAs (lncRNAs), such as NONRATT021972, may regulate pain signaling pathways and are potential therapeutic targets.^664^ The level of serum NONRATT021972 is positively correlated with the degree of neuropathic pain in T2DM patients. Inhibiting NONRATT021972 may relieve the symptoms of neuropathic pain in T2DM patients.^665^
- **Neuronal damage markers:** Elevated serum neuron-specific enolase (NSE) levels reflect neuronal injury and correlate with DPN severity. The serum NSE level in DPN patients is significantly higher than that in diabetes patients without neuropathy, and the serum NSE level is related to the severity or lesion stage of DPN.^666^ Increased serum phosphorylated neurofilament heavy chain (pNF-H) is associated with SFN in patients with impaired glucose tolerance.^667^

Current treatments for DN primarily focus on symptom management, lacking effectiveness in reversing or halting disease progression. Emerging gene therapy and cell-based regenerative treatments face considerable challenges in delivery and specificity. Targeted delivery to peripheral nerves remains challenging due to the blood-nerve barrier, and off-target effects can pose safety risks. Future research should focus on vector development for enhanced specificity and safety and optimize CRISPR-based approaches for long-term gene editing in DN-related genes. Moreover, although preclinical studies have yielded promising results, large-scale, double-blinded clinical trials remain necessary to verify the therapeutic efficacy and feasibility of these approaches in DN patients.

## Future directions for clinical management and patient care

### Personalized medicine

Personalized medicine, leveraging genomics, metabolomics, and other multi-omics approaches, holds significant promise for the early diagnosis and therapeutic monitoring of DN. Integrating biomarkers into clinical practice can tailor interventions, leading to more effective management strategies. Genetic variants influencing drug metabolism can facilitate the selection of pharmacological treatments, improving efficacy and reducing adverse effects. For instance, pharmacogenomic testing can identify patients more likely to respond to specific analgesics or may be at risk for adverse reactions.^668^ This approach enhances therapeutic outcomes by aligning treatments with the patient’s genetic makeup. Recent studies have highlighted the utility of biomarkers, such as NGF and BDNF, in predicting the progression of neuropathy and response to therapy. Elevated levels of NGF and BDNF have been associated with nerve regeneration and repair mechanisms.^669,670^ Metabolomics offers insights into metabolic alterations associated with DN. Metabolic profiling can identify specific patterns linked to neuropathy, facilitating early detection and personalized interventions.^671^

Additionally, catalyzing DPN discoveries through bioinformatics is crucial due to the complex pathophysiology of DPN. Integrative methods combining two or more Omics datasets can identify novel associations in DPN, and computational methods can be leveraged to identify drug targets.^672,673^ Transcriptomics research analyzes gene expression changes in peripheral nerves of both DPN animal models and humans, consistently revealing immune response and inflammatory pathway involvement in neuropathy. Single-cell RNA sequencing and spatial transcriptomics are novel approaches that provide deeper insights into the cellular heterogeneity in DPN pathogenesis.^674–676^ Integrating the vast knowledge from Omics datasets with machine learning and deep learning will be crucial in DPN research to identify therapeutic targets.^671^

### Multidisciplinary management

A multidisciplinary approach involving endocrinologists, neurologists, podiatrists, pain specialists, and rehabilitation therapists is crucial for the comprehensive management of DN patients. Collaborative care models enhance lifestyle interventions by focusing on diet, exercise, and foot care, essential for preventing complications such as diabetic foot ulcers. Evidence suggests that integrated care pathways lead to improvements, including reduced hospitalizations and enhanced quality of life.^591,677^ Multidisciplinary teams address various aspects of DN, such as glycemic control, neuropathic pain management, and prevention of secondary complications. For instance, regular foot examinations conducted by podiatrists can prevent ulcerations and amputations by identifying and treating problems early.^678^ Rehabilitation specialists provide tailored exercise programs to improve nerve function and reduce pain, with physical therapy shown to enhance balance, strength, and overall mobility in DN patients.^679^ Additionally, dietitians assist in nutritional management, optimizing metabolic control, and potentially slowing disease progression.

### Digital health and remote monitoring

Advancements in digital health technologies, such as wearable devices and telemedicine, offer promising avenues for long-term patient management. These technologies facilitate continuous monitoring of patients’ health status, enabling timely interventions and personalized care plans. Wearable devices can monitor parameters like blood glucose levels and physical activity and even detect early signs of foot ulcers through temperature and pressure sensors.^680^ Smart insoles and socks with sensors can alert patients and healthcare providers to areas of high pressure or temperature changes indicative of tissue damage.^681^ Telemedicine provides remote access to healthcare services, which is especially beneficial for patients with limited mobility or those living in remote areas. Telehealth interventions have been shown to improve glycemic control and patient satisfaction.^682^ Mobile Health Applications facilitate self-management by providing educational resources, medication reminders, and tools for tracking symptoms and glucose levels.^683,684^ Advanced imaging techniques, such as high-resolution MRI and positron emission tomography, offer potential for early DN detection and disease progression monitoring.^685,686^

### AI in DN management

AI and machine learning technologies are increasingly being integrated into healthcare, offering new opportunities for the management of DN. Early Detection and Diagnosis: AI algorithms can analyze large datasets to identify patterns indicative of early neuropathic changes. Machine learning models have been developed to predict the onset of DN by processing clinical variables, genetic information, and imaging data.^687^ For example, deep learning techniques applied to NCS and skin biopsy images can enhance diagnostic accuracy.^688^ AI can assist in stratifying patients based on their risk of developing complications. Predictive analytics can consider many factors, including glycemic control, comorbidities, and lifestyle behaviors, to forecast disease progression and personalize intervention strategies.^689^ Decision support tools utilizing AI can help clinicians select the most effective therapies, adjust dosages, and anticipate potential side effects.^689,690^ AI-powered applications enhance remote monitoring by analyzing data from wearable devices and alerting healthcare providers to significant changes. Natural language processing enables virtual assistants and chatbots to support patients.^690^

Despite the potential benefits, challenges include data privacy concerns, the need for large and diverse datasets to train algorithms, and ensuring the transparency and interpretability of AI models remain.^691,692^ Ethical considerations and regulatory frameworks must evolve alongside technological advancements to ensure patient safety and trust. Bioinformatics and multi-omics approaches are pivotal in uncovering the complex pathophysiological mechanisms of DN, facilitating novel therapeutic targets. However, addressing the associated challenges is essential to fully realize the benefits of AI in DN management.

## Conclusions

Despite advancements in our understanding of DN, addressing the growing healthcare burden posed by this complication of diabetes remains challenging. A paradigm shift is occurring, with research focusing on a more comprehensive understanding of nerve metabolism, including the interactions between glucose, lipids, and bioenergetics in the PNS. The focus of preclinical studies has shifted from investigating glucose metabolism alone to exploring more complex mechanisms, such as the metabolic interplay between Schwann cells and axons and the role of insulin resistance in nerve function.

Further research remains warranted to address unanswered questions, such as whether DN results from global metabolic reprogramming of the PNS and how energy transfer or toxic byproducts between nerve cells contribute to the disease. Addressing these questions will aid in developing therapies that target the underlying mechanisms of DN.

The rising prevalence of diabetes and obesity, coupled with DN’s severe impact on quality of life—including pain, disability, and increased risk of amputations—necessitate effective treatment. The next decade will be pivotal in translating accumulating clinical and preclinical findings into meaningful therapies that can prevent or reverse the course of DN, ultimately improving outcomes for millions of patients.
